# Epigenetic activation of secretory phenotypes in senescence by the FOXQ1-SIRT4-GDH signaling

**DOI:** 10.1038/s41419-023-06002-9

**Published:** 2023-07-29

**Authors:** Xinpei Sun, Qian Li, Yunyi Tang, Wanjin Hu, Gengyao Chen, Hongguang An, Daoyuan Huang, Tanjun Tong, Yu Zhang

**Affiliations:** 1 Beijing Key Laboratory of Protein Posttranslational Modifications and Cell Function, Department of Biochemistry and Biophysics, School of Basic Medical Sciences, Peking University Health Science Center, Beijing, 100191 China; 2 Department of Orthodontics, Peking University School and Hospital of Stomatology, National Engineering Laboratory for Digital and Material Technology of Stomatology, Beijing Key Laboratory of Digital Stomatology, 100081 Beijing, China

**Keywords:** Epigenetics, Senescence

## Abstract

Although metabolic reprogramming is characterized as a hallmark of aging, implications of the crucial glutamate dehydrogenase (GDH) in human senescence remain poorly understood. Here, we report that GDH activity is significantly increased in aged mice and senescent human diploid fibroblasts. This enzymatic potentiation is associated with de-repression of GDH from its functionally suppressive ADP-ribosylation modification catalyzed by NAD-dependent ADP-ribosyltransferase/deacetylase SIRT4. A series of transcription analyses led to the identification of FOXQ1, a forkhead family transcription factor (TF), responsible for the maintenance of SIRT4 expression levels in juvenile cells. However, this metabolically balanced FOXQ1-SIRT4-GDH axis, is shifted in senescence with gradually decreasing expressions of FOXQ1 and SIRT4 and elevated GDH activity. Importantly, pharmaceutical inhibition of GDH suppresses the aberrantly activated transcription of *IL-6* and *IL-8*, two major players in senescence-associated secretory phenotype (SASP), and this action is mechanistically associated with erasure of the repressive H3K9me3 (trimethylation of lysine 9 on histone H3) marks at *IL-6* and *IL-8* promoters, owing to the requirement of α-ketoglutaric acid (α-KG) from GDH-mediated glutamate dehydrogenase reaction as a cofactor for histone demethylation. In supplement with the phenotypic evidence from FOXQ1/SIRT4/GDH manipulations, these data support the integration of metabolism alterations and epigenetic regulation in driving senescence progression and highlight the FOXQ1-SIRT4-GDH axis as a novel druggable target for improving human longevity.

## Introduction

One of the central themes characterizing cellular senescence is the alteration of nutrient sensing and metabolic reprogramming [1]. These metabolic changes are broadly intertwined with other hallmarks of aging including loss of proteostasis, defective intercellular communication and epigenetic deregulation to affect the homeostasis of aged cells and to cause their final irreversible growth arrest and functional exhaustion [1]. The intimate link between metabolism and epigenetics is rooted in the utilization of metabolic intermediates as substrates or cofactors in DNA/histone modifying enzyme-mediated biochemical reactions on chromatin [2]. Deregulated production of these intermediates, best exemplified as S-adenosyl methionine (SAM) for DNA and histone methylation [3], and α-KG for dioxygenase-catalyzed demethylation [4], is emerging as driving force in a wide spectrum of human pathologies. Notably, genetic mutations of the isocitrate dehydrogenase 1 and 2 (IDH1/2) enzymes, accounting for aberrant production of 2-hydroxyglutarate (2HG) from α-KG, are critically implicated in a majority of subtypes of malignant gliomas and leukemia [5, 6], suggesting an important role of α-KG accompanying TET-family and JMJC-family dioxygenases in DNA and histone demethylation for the maintenance of normal cell growth and activities [6].

The glutamate dehydrogenase (GDH) in mammals catalyzes the reversible interconversion between glutamate and α-ketoglutarate, and thus is implicated in carbon and nitrogen metabolism, redox and acid-base homeostasis, and biosynthesis of lipid and lactate showing both catabolic and anabolic functionalities [7]. Due to the importance of glutamine/glutamate requirement in specific tissues, and the roles of glutamate as neurotransmitters and neurotoxins [8], glutamate homeostasis involving the catalytic activity of GDH has been emphasized in several aging-related disorders, such as Parkinson’s disease [9]. However, whether and how nutrition/metabolism-independent epigenetic function of GDH is implicated in aging/senescence remain elusive.

Senescence-associated secretory phenotype (SASP) is an emerging biomarker of cellular senescence [10]. This evolutionally conserved pro-inflammatory secretome not only reinforces senescence progression in a cell-autonomous manner, but also provokes system-wide response through dysfunctional immunosurveillance in many age-related pathological conditions [1]. Therefore, pharmacological intervention of SASP by targeting its drivers or key components is rising as a promising therapeutic strategy for healthy aging [11]. As a key transcription factor driving the expression of a large cohort of pro-inflammatory genes, NF-κB is also a master regulator of SASP [12]. Interestingly, α-KG-dependent histone demethylases critically contribute to NF-κB-mediated inflammatory gene transcription through erasing repressive histone modifications at promoters/enhancers [13], pointing to a potential linkage between GDH-catalyzed α-KG production and SASP induction.

In our effort to identify molecular pathways in relation to metabolic reprogramming of senescence, we found increased GDH activity in aged mice and cellular senescence models. These changes were attributed to the decrease of suppressive ADP-ribosylation modification of GDH catalyzed by SIRT4. Through a combination of transcription analysis approaches, we identified the FOXQ1-SIRT4-GDH axis as a pivotal cascade to integrate metabolic environment and epigenetic regulation of GDH activity and secretory phenotypes in senescence.

## Results

### Glutamate dehydrogenase activity is increased in aging and cellular senescence

**Fig. 1:**
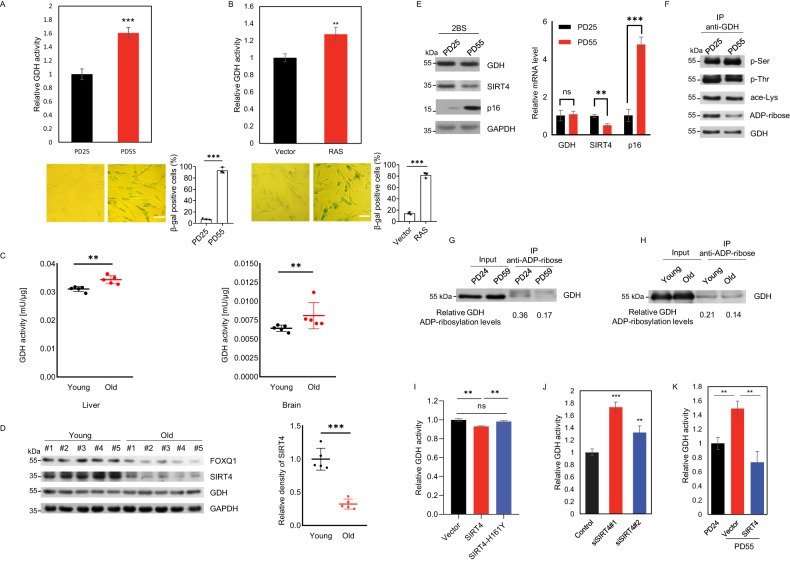
Glutamate dehydrogenase activity is increased in aging and cellular senescence. **A** GDH activity of 2BS cells at PD25 or PD55 was measured by GDH Activity Assay Kit and demonstrated as the ratio relative to averaged PD25 result (top). Representative images of SA-β-gal staining are shown (bottom). Bar represents mean±S.D. for triplicates. Scale bar, 100 μm. **B** Ras-induced senescent IMR90 cells were subjected to GDH activity examination (top). Representative images of SA-β-gal staining are shown (bottom). Bar represents mean±S.D. for triplicates. Scale bar, 100 μm. **C** GDH activity was determined in liver and brain tissues of young (3 months) and old BALB/c mice (26 months). Each point in the scatter plot represents a mouse, and the line represents group means±S.D. (*n* = 5). **D** Protein levels of GDH, SIRT4 and FOXQ1 in brain tissues of young (3 months) and old (26 months) BALB/c mice (*n* = 5). GAPDH was used as a loading control (left). Densitometric analysis of the relative density of SIRT4 normalized with GAPDH (right). **E** Protein and mRNA levels of GDH, SIRT4 and p16 in 2BS cells with indicated PDs. Bar represents mean±S.D. for triplicates. **F** GDH proteins were immunoprecipitated from 2BS cells at PD25 or PD55. GDH protein modifications were then determined by western blotting analysis with the anti-pan-phospho-serine (p-Ser), -phospho-threonine (p-Thr), -acetyl-lysine (ace-Lys), and -ADP-ribosylation (ADP-ribose) antibodies. **G**, **H** Reciprocal immunoprecipitation in 2BS cells and livers of young and old mice was performed with the use of anti-ADP-ribose antibody. GDH proteins with ADP-ribosylation modification were then probed by anti-GDH antibody conjugated with TrueBlot® Secondary Antibodies (HRP). GDH ADP-ribosylation levels indicated below were quantified as pull-downed band density relative to the relevant input. **I** HEK293 cells were transfected with indicated plasmids. 48 h after transfection, cell lysates were analyzed for GDH activity. Bar represents mean±S.D. for triplicates. **J** HEK293 cells transfected with siRNA against SIRT4 or control were subjected to GDH activity examination. Bar represents mean±S.D. for triplicates. **K** GDH activity was measured in 2BS cells infected with indicated lentivirus. Bar represents mean±S.D. for triplicates. For statistical comparisons in (**A**–**E**) and (**J**), statistical significance was calculated using two-tailed Student’s *t* test. For (**I**) and (**K**), one-way ANOVA was used. ^*^P < 0.05; ^**^P < 0.01; ^***^P < 0.001.

Glutamate dehydrogenase (GDH) catalyzes the oxidative deamination of glutamate to α-ketoglutarate [7] - one of the rate-determining intermediates in tricarboxylic acid (TCA) cycle in mitochondria [14]. In our attempt to understand aging-associated metabolic reprogramming, we identified an elevation of GDH activity in cellular senescence. With human embryonic lung diploid fibroblast 2BS cells in a long-term culturing, we observed most of the cells were presented with enlarged and flattened morphology, and were positive for senescence-associated β-galactosidase (SA-β-gal) activity with population doubling (PD) of 55 compared to PD25, indicating a replicative senescent state at PD55 (Fig. 1A). Importantly, GDH activity examination by an enzymatic assay suggested that senescent 2BS cells showed a significant elevation of GDH activity (Fig. 1A). Concordantly, we observed a significant increase of GDH activity in oncogenic RAS-induced senescent (OIS) IMR90 fibroblasts, accompanied by induced senescent phenotypes indicated by SA-β-gal staining (Fig. 1B). As a control, the activity of citrate synthase (CS) – a key enzyme in TCA cycle serving as mitochondrial content marker [15] – showed no significant changes after normalization (Supplemental Fig. 1A).

To test whether this observation of increased GDH activity in cellular senescence could be recapitulated in aged animals, we prepared fresh protein lysates from liver and brain in a paired group of young and old BALB/c mice, given the significant expression of GDH in these tissues [16]. Examination with these extracts indicated similarly increased GDH activity but preserved CS activity in old mice compared with young tissues (Fig. 1C and Supplemental Fig. 1C), suggesting a consistent upward regulation of GDH functionality in both cellular senescence and aging. Interestingly, brain samples separately prepared from cerebral cortex, hippocampus, and striatum on basis of their distinct metabolic features and pathologic relevance [17], showed a specific increase of GDH activity in aged cortex (Supplemental Fig. 1D), where Alzheimer’s disease primarily manifests .

### Decreased GDH ADP-ribosylation is accompanied by SIRT4 decline in aged cells

To understand the molecular mechanism underlying this age-associated up-regulation of GDH activity, we first determined whether the protein level of GDH changed in senescence and aging. However, no discernible changes were observed in either senescent cells or aged mice (Fig. 1D, E and Supplemental Fig. 1F). We then asked whether GDH was subjected to post-translational modification-associated activity changes in aging. For this purpose, we immunoprecipitated GDH protein from 2BS cells at early and late PDs and examined its possible post-translational modifications by use of specific antibodies against pan-phosphorylation (serine or threonine) and pan-acetylation (Fig. 1F). The results demonstrated that there were no discernible changes of GDH phosphorylation or acetylation along with increased PDs.

Interestingly, previous investigations have demonstrated that SIRT4 exhibits mono-ADP-ribosylation activity and catalyzes the ADP-ribosylation modification of GDH [18]; and overexpression of SIRT4 to induce GDH ADP-ribosylation could repress the enzymatic activity of GDH and limit the downstream metabolism of glutamate and glutamine to generate ATP, while loss of SIRT4 activates GDH [18, 19]. We thus probed this modification, and the results indicated a significantly lower degree of ADP-ribosylation observed in senescent compared to the young 2BS cells, implying a specific involvement of GDH ADP-ribosylation in modulating GDH activity in senescence and aging (Fig. 1F). Consistently, a reciprocal immunoprecipitation using anti-ADP-ribosylation antibody indicated that less GDH was precipitated from PD59- in comparison to PD24-2BS cells (Fig. 1G). Similar results were obtained in mouse liver and brain samples (Fig. 1H and Supplemental Fig. 1G).

Mitochondrial GDH has been widely reported to be ADP-ribosylated by SIRT4 and ADP-ribosylated GDH showed decreased glutamate dehydrogenase activity [20]. Hence, to determine whether the increased GDH activity in senescence is associated with altered SIRT4 expressions, we measured mRNA and protein levels of SIRT4 by quantitative reverse transcription PCR (RT-qPCR) and western blotting respectively in 2BS cells at different PDs. The results showed a significant decrease of SIRT4 expression in senescent compared to young cells at both mRNA and protein levels (Fig. 1E), correlating with decreased ADP-ribosylation of GDH. Furthermore, western blotting analysis demonstrated decreased expression of SIRT4 in brain and liver tissues of old mice compared to the young ones (Fig. 1D and Supplemental Fig. 1F), implying a potential common SIRT4-dependent regulatory theme for increased GDH activity in senescence and aging.

To substantiate the involvement of SIRT4 in functional regulation of GDH, and also according to the inverse correlation between SIRT4 expression and GDH activity in *Sirt4* genetically ablated mice [18], we transfected wild-type or the catalytically inactive H161Y mutant SIRT4 deficient for ADP-ribosyltransferase activity [19] into HEK293 cells. Examination of GDH activity revealed that cells overexpressing wild-type SIRT4 but not the catalytically inactive mutant led to decreased GDH activity (Fig. 1I). In contrast, knockdown of SIRT4 resulting in significant increase of GDH activity in HEK293 cells (Fig. 1J). We then asked whether the age-related GDH activation was associated with SIRT4 expression changes. Indeed, supplementation of decreased SIRT4 expression in senescent 2BS cells with lentiviral infection attenuated the up-regulated GDH activity (Fig. 1K). These results suggested that down-regulation of SIRT4 was critical for the above observed activation of GDH in cellular senescence.

### FOXQ1 activates SIRT4 transcription by directly binding to its promoter

**Fig. 2:**
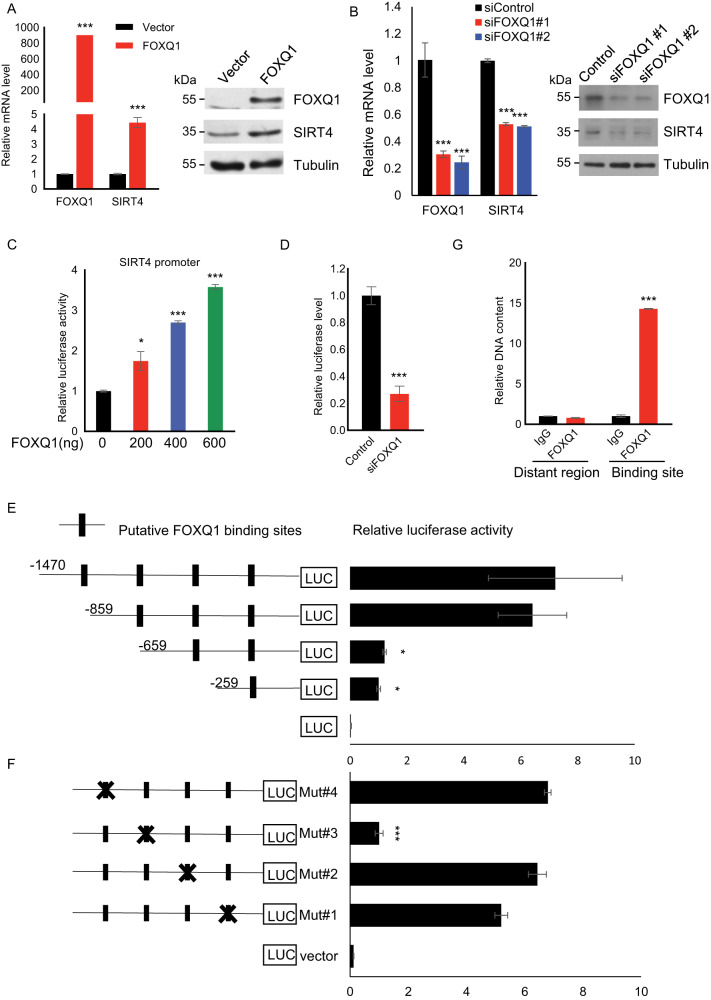
FOXQ1 activates SIRT4 transcription by directly binding to its promoter. **A** HEK293 cells were transfected with empty vector or FOXQ1 expression constructs. The mRNA or protein level of SIRT4 and FOXQ1 in these cells was measured by RT-qPCR (left) or western blotting (right). Bar represents mean±S.D. for triplicates. **B** HEK293 cells were transfected with siRNAs against FOXQ1 or control. The mRNA or protein level of SIRT4 and FOXQ1 in these cells was measured by RT-qPCR (left) or western blotting (right). Bar represents mean±S.D. for triplicates. **C** HEK293 cells cultured in 24-well plates were transfected with FOXQ1 of indicated concentration together with a SIRT4 promoter construct (pGL3-SIRT4) for 48 h. Luciferase activity was then measured with a dual luciferase kit and the data was shown as relative value to the control readout. Bar represents mean±S.D. for triplicates. **D** Relative luciferase activity of pGL3‐SIRT4 in HEK293 cells transfected with siRNAs against FOXQ1 or control. Bar represents mean±S.D. for triplicates. **E** Normalized luciferase activities in HEK293 cells transfected with a series of indicated truncations of SIRT4 promoter together with FOXQ1 expression plasmid. Bar represents mean±S.D. for triplicates. **F** Normalized luciferase activities in HEK293 cells transfected with a series of SIRT4 promoters with indicated point mutations at the putative FOXQ1 binding sites together with FOXQ1 expression plasmid. Bar represents mean±S.D. for triplicates. **G** ChIP analysis on the recruitment of endogenous FOXQ1 at SIRT4 promoter or a distal region serving as negative control. Bar represents mean±S.D. for triplicates. For statistical comparisons in (**A**–**F**),^*^P < 0.05; ^**^P < 0.01; ^***^P < 0.001 (student’s *t* test).

We next sought to explore the molecular mechanisms accounting for the decline of SIRT4 expression in senescence. In this regard, we noted that an intersection between the set of predicted transcription factors for SIRT4 through TFSEARCH software [21] and the fact of direct binding of forkhead family transcription factor (TF) FOXQ1 at SIRT4 promoter according to a published ChIP-seq (chromatin immunoprecipitation coupled with massively parallel DNA sequencing) analysis in the LoVo human colon cancer cells [22] suggested that FOXQ1 could be a putative candidate TF for transcription regulation of SIRT4. To determine the effect of FOXQ1 on SIRT4 expression, FOXQ1 was either overexpressed or knocked down in HEK293 cells, followed by the examination of FOXQ1 and SIRT4 expression levels using RT-qPCR and western blotting. The results showed that gain-of-function for FOXQ1 resulted in increased SIRT4 expression in both mRNA and protein levels, whereas loss-of-function for FOXQ1 caused a significant decrease of SIRT4 expression (Fig. 2A, B).

To further identify the *cis*-regulatory element at SIRT4 promoter responsible for FOXQ1-dependent SIRT4 transcription regulation, a SIRT4 promoter-driven luciferase reporter harboring the 1470 bp fragment upstream of SIRT4 transcription start site (TSS) and FOXQ1 expression construct were co-transfected in HEK293 cells to assess the effect of FOXQ1 on SIRT4 promoter activity. This dual luciferase assay indicated that overexpression of FOXQ1 could activate SIRT4 promoter in a dose-dependent manner (Fig. 2C). Concordantly, FOXQ1 depletion led to a significant decrease of SIRT4 promoter activity (Fig. 2D). With a series of truncated SIRT4 promoter constructs, we found the reporter with −1470 – −659 portion deleted showed most significantly decreased SIRT4 promoter activity that induced by FOXQ1 (Fig. 2E). The luciferase reporter assays with site-directed SIRT4 promoter point mutations suggested that the predicted FOXQ1 binding site at around −816 – −819 was critical for FOXQ1-induced transactivation of the SIRT4 promoter (Fig. 2F). Indeed, ChIP analysis further validated the direct recruitment of endogenous FOXQ1 at SIRT4 promoter in 2BS cells (Fig. 2G). These data together suggested that FOXQ1 could function as a transcription factor of SIRT4 by directly binding to its promoter.

### Loss of FOXQ1-maintained SIRT4 transcription in senescence

**Fig. 3:**
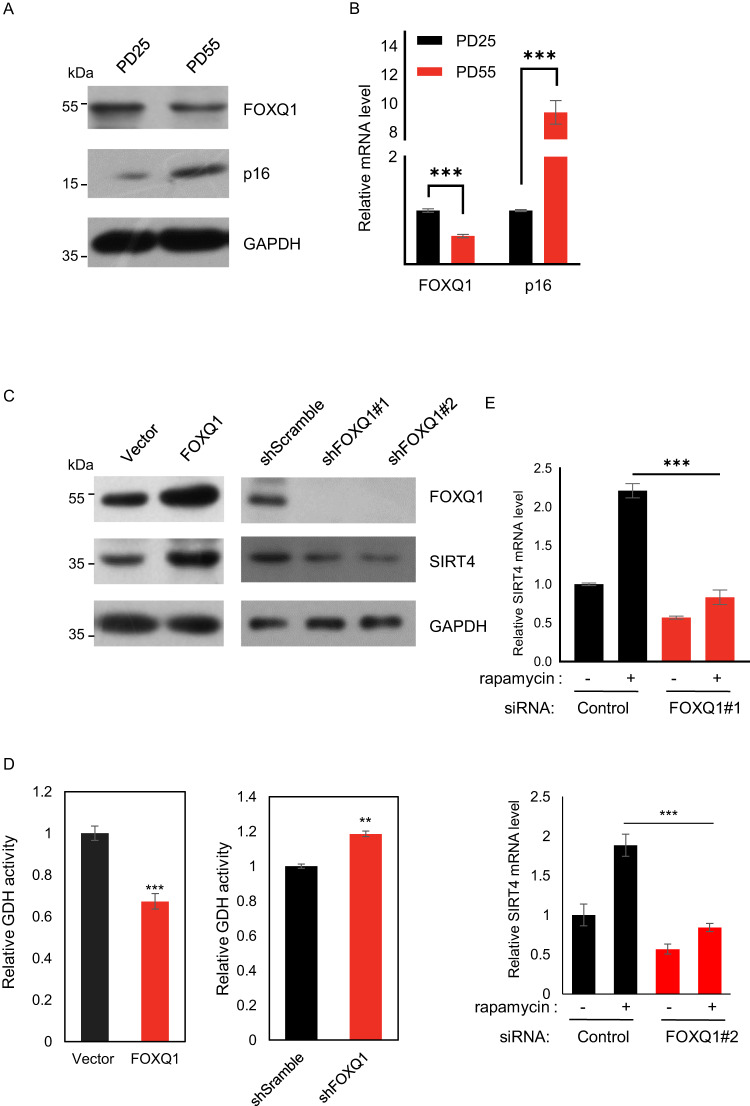
Loss of FOXQ1-maintained SIRT4 transcription in senescence. **A** Western blot analysis of indicated genes' protein levels in replicative senescence. **B** RT-qPCR analysis on the mRNA levels of indicated genes in replicative senescence. Bar represents mean±S.D. for triplicates. **C** 2BS cells were infected with lentivirus expressing FOXQ1 or control (left), or short hairpin RNAs (shRNAs) against FOXQ1 or scramble control (right), and maintained for one month of selection. Western blotting was then performed to analyze the protein levels of indicated genes. (**D**) Relative GDH activity of the cells in (**C**). Bar represents mean±S.D. for triplicates. **E** HEK293 cells were transfected with siRNAs against FOXQ1 or control and harvested after rapamycin treatment (100 nM, 12 h). SIRT4 mRNA level was then analyzed by RT-qPCR. Bar represents mean±S.D. for triplicates. For statistical comparisons in (**B**–**E**), ^*^P < 0.05; ^**^P < 0.01; ^***^P < 0.001 (student’s *t* test).

To probe the changes of FOXQ1/SIRT4 axis in cellular senescence, we first compared their expression levels in replicative exhaustion. In contrast to the increase of p16 protein levels as a molecular marker of senescence [23], FOXQ1 expression level was significantly decreased in aged 2BS cells (Fig. 3A, B). To test whether FOXQ1 played a causal role in transcriptional regulation of SIRT4 in 2BS cells, we transduced 2BS cells at early passage with lentivirus driving FOXQ1 overexpression or knockdown. Consistently, the expression of SIRT4 positively responded to FOXQ1 levels (Fig. 3C). Importantly, in line with decreased SIRT4 underlying the de-repression of GDH activity in senescence (Fig. 1K), the enzymatic activity of GDH was decreased in FOXQ1 stably integrated 2BS cells and concordantly increased in FOXQ1 depleted cells (Fig. 3D), implying a consistent regulatory theme for the FOXQ1/SIRT4/GDH axis in different scenarios.

The expression levels of SIRT4 have been reported to be increased under rapamycin-mediated mTORC1 inhibition [21]. Surprisingly, RT-qPCR analysis indicated that FOXQ1 removal nearly abolished rapamycin-induced transcriptional activation of SIRT4 (Fig. 3E). Given the tight connection between metabolism and aging [1], these data demonstrated a potential implication of FOXQ1-mediated transcriptional regulation of SIRT4 in cellular senescence.

### GDH inhibition attenuates the secretory phenotypes in senescence progression

**Fig. 4:**
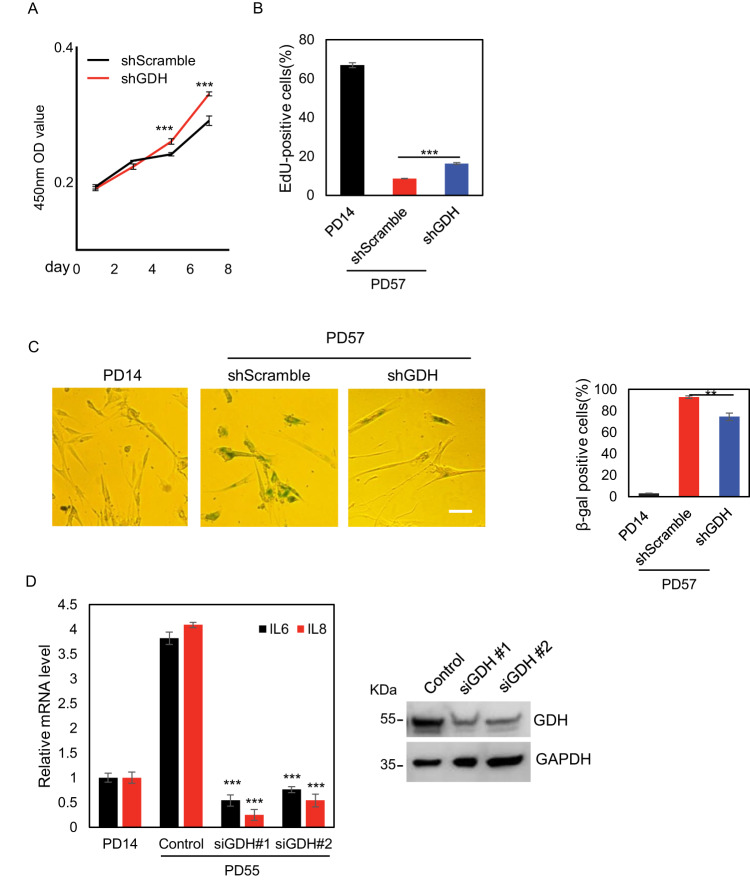
GDH Inhibition Attenuates the Secretory Phenotypes in Senescence Progression. **A** Growth curves of 2BS cells infected with lentivirus for GDH knocking down were determined by CCK-8 assay. Data were represented as the mean±S.D. for triplicates. **B** EdU incorporation was performed in 2BS cells with the indicated treatment. Percentage of positive cells was shown. Bar represents mean±S.D. for triplicates. **C** SA-β-gal analysis in 2BS cells with the indicated PDs and treatment. Bar represents mean±S.D. for triplicates. Representative immunofluorescence images were shown on the left. Scale bar, 100 μm. **D** RT-qPCR analysis on the mRNA levels of indicated genes in 2BS cells transfected with siRNAs against GDH or control (left). The protein level of GDH was measured by western blotting (right). For statistical comparisons in (**A**–**D**), ^*^P < 0.05; ^**^P < 0.01; ^***^P < 0.001 (student’s *t* test).

Cellular senescence is characterized by an array of diverse phenotypes linked to the functional exhaustion of aged cells, we next asked how the altered GDH activity contributed to senescence progression. For this purpose, we employed both RNAi-based knockdown (short hairpin RNA, shRNA) and pharmacological inhibitors to interfere with the function of GDH. Interestingly, GDH depletion promoted cell growth, as demonstrated by the increased cell proliferation and EdU incorporation compared to the control cells (Fig. 4A, B, Supplemental Fig. 2A, B). Notably, this interference could partially ameliorate senescence progression, indicated by the decreased SA-β-gal staining of senescent 2BS cells (Fig. 4C). We further evaluated the effect of GDH knockdown on *IL-6* and *IL-8* induction, two major components of senescence-associated secretory phenotype (SASP), which is well recognized in initiating inflammatory responses in a broad range of age-related phenotypes and pathologies [24]. Consistently, GDH knockdown markedly suppressed the mRNA levels of *IL-6* and *IL-8* in senescent 2BS cells (Fig. 4D), while no significant changes of the anti-inflammatory cytokine *IL-10* were observed (Supplemental Fig. 2C).

Next, we treated 2BS cells with 2-allyl-1-hydroxy-9,10-anthraquinone (R162) or green tea polyphenol epigallocatechin gallate (EGCG), both of which could function as small molecular inhibitors of GDH [25, 26]. Their efficacy in GDH inhibition was validated in our system (Supplemental Fig. 2D and E). In line with GDH knockdown associated phenotypic changes, both R162 and EGCG addition partially ameliorated senescence progression, as indicated by the decreased SA-β-gal staining, increased EdU incorporation and cell proliferation in treated compared to control 2BS cells at late passages (Supplemental Fig. 2H–K). Furthermore, the mRNA levels of *IL-6* and *IL-8* but not *IL-10* showed moderate but significant reduction upon either treatment in senescent 2BS cells (Supplemental Fig. 2F, G). Consistently, aged mice treated with EGCG also exhibited decreased *IL-6* and *IL-8* as indicated by ELISA, immunohistochemistry and western blotting analyses (Supplemental Fig. 2L–N). The senescence relieving effect by GDH inhibitor R162 and EGCG was also validated in RAS-induced OIS model in IMR90 fibroblast cells (Supplemental Fig. 2O–Q). To determine whether *IL-6* and *IL-8* play an essential role in GDH-affected senescence progression, we performed SA-β-gal staining and EdU incorporation assay in shRNA-mediated GDH knockdown and control cells together with the treatment of IL-6 and IL-8. The results implied that administration of *IL-6* and *IL-8* enhanced senescence phenotypes in the control cells as revealed by higher percentage of SA-β-gal positive cells and lower proportion of EdU positive cells compared with those lacking the treatment of IL-6 and IL-8 (Supplemental Fig. 2R, S). Importantly, the senescence relieving effect induced by GDH depletion was partially reversed by IL-6/IL-8 supplementation (Supplemental Fig. 2R, S), further supporting their contributions to GDH-regulated senescence progression. Taken together, these observations suggested that GDH inhibition or depletion could negatively regulate senescence progression with the associated *IL-6* and *IL-8* induction partially suppressed.

### Increased α-ketoglutarate production potentiates histone/DNA demethylation at key inflammatory gene promoters in senescence

**Fig. 5:**
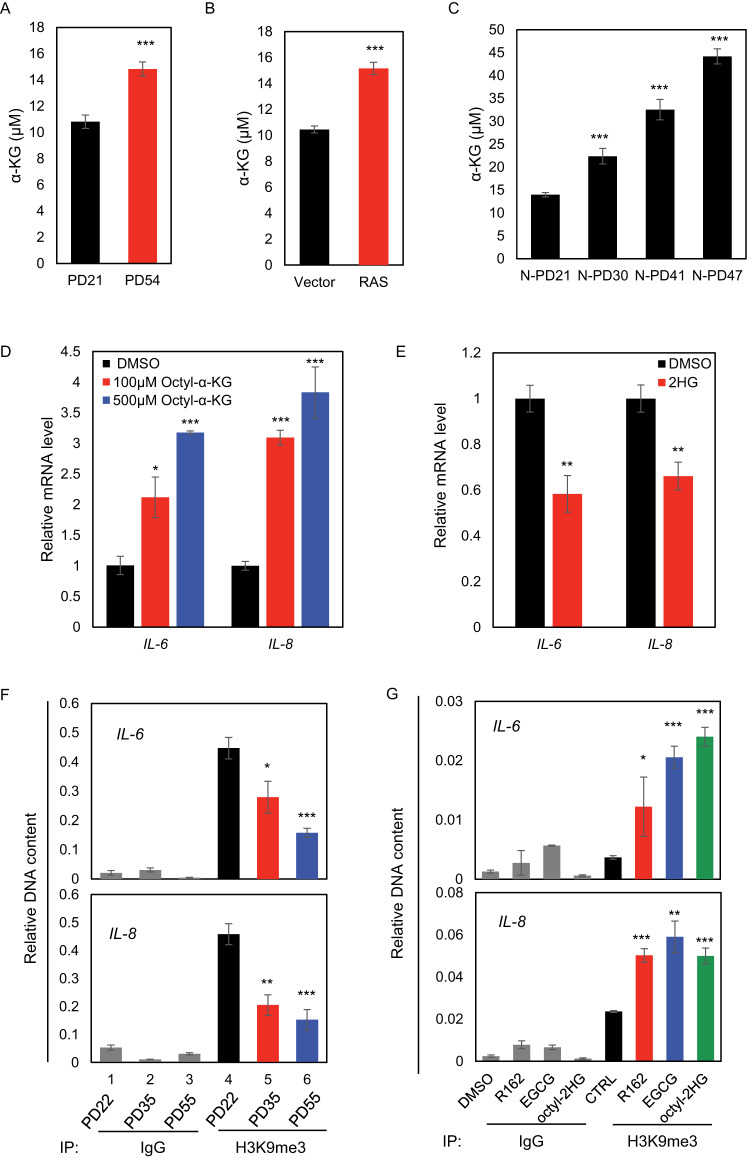
Increased α-ketoglutarate production potentiates histone/DNA demethylation at key inflammatory gene promoters in senescence. **A** Intracellular α-KG levels were determined in the young and senescent 2BS cells. Bar represents mean±S.D. for triplicates. **B** Intracellular α-KG levels were determined in IMR90 cells with the indicated infections. Bar represents mean±S.D. for triplicates. **C** Nuclear α-KG levels were determined by using isolated nuclei of 2BS cells at the indicated PDs. Bar represents mean±S.D. for triplicates. **D** RT-qPCR analysis on the mRNA levels of indicated genes in 2BS cells (PD14) exposed to DMSO or octyl-α-KG. Bar represents mean±S.D. for triplicates. **E** RT-qPCR analysis on the mRNA levels of indicated genes in 2BS cells (PD55) exposed to DMSO or octyl-2HG. Bar represents mean±S.D. for triplicates. **F** ChIP analysis on the enrichment pattern of H3K9me3 at *IL-6* and *IL-8* promoters in 2BS cells at different PDs. The normal IgG antibody was used as negative control. Bar represents mean±S.D. for triplicates. **G** ChIP analysis on the enrichment pattern of H3K9me3 at *IL-6* and *IL-8* promoters in 2BS cells under the indicated treatment. Bar represents mean±S.D. for triplicates. For statistical comparisons in (**A**–**G**), ^*^P < 0.05; ^**^P < 0.01; ^***^P < 0.001 (student’s *t* test).

The α-KG produced in GDH catalyzed oxidative deamination of glutamate is not only a key metabolite in central carbon metabolism, but also a critical enzymatic co-factor for a large family of dioxygenases [4]. This importance has been demonstrated in many malignancies, where 2-hydroxyglutarate as structural analogue of α-KG, could competitively inhibit α-KG dependent enzymes, including histone demethylases, and drive epigenetic aberrances and oncogenesis [27]. Along with the increased GDH activity in senescence, intracellular α-KG levels in both late passage 2BS cells and oncogenic RAS-induced senescent IMR90 cells were higher than those in young ones (Fig. 5A, B). Interestingly, evaluation of the isolated nuclei showed much increased fold changes of nuclear α-KG abundance when comparing cells from late versus early passages, implying a greater impact of GDH on gene transcription through nuclear α-KG availability in histone/DNA demethylation (Fig. 5C). This point was further supported by the increased α-KG/succinate ratio in both senescent 2BS and IMR90 cells (Supplemental Fig. 3A–C), which is better indicator for α-KG amenable to α-KG-dependent dioxygenases. We then confirmed the alteration of α-KG during aging in animal model, which demonstrated that aged mice contained significantly higher amount of α-KG in both liver and brain compared with the young mice (Supplemental Fig. 3D, E).

On the other hand, treatment of 2BS cells with the membrane permeable octyl-α-KG at early passage (PD14) resulted in increased *IL-6/IL-8* mRNA levels (Fig. 5D), in line with our observations of the elevated nuclear α-KG abundance (Fig. 5C) and the positive regulation of *IL-6/IL-8* expressions by GDH in senescence (Fig. 4D). In contrast, senescent cells treated with 2HG showed significant reduction of both *IL-6* and *IL-8* mRNA levels (Fig. 5E). Consistent with the repressive role of H3K9me3 in gene transcription and the transcriptional activation of SASP genes in senescence [10, 24], ChIP analysis suggested that the enrichment of H3K9me3 around *IL-6* and *IL-8* promoters was strikingly reduced in senescent 2BS cells (Fig. 5F). Importantly, exposure of 2BS cells to both GDH inhibitors or 2HG could reverse this reduction, suggesting that GDH-associated α-KG production was crucial for the removal of repressive H3K9me3 marks on SASP gene promoters in senescence (Fig. 5G). Concordantly, application of R162 or EGCG suppressed the relevant tendency of increasing α-KG/succinate ratio (Supplemental Fig. 3G). Moreover, EGCG treatment to the aged mice also resulted in decreased α-KG abundance (Supplemental Fig. 3D, E). Interestingly, a further investigation by co-treatment with the IKK/NF-κB inhibitor BAY 11-7082 revealed a beneficial effect of α-KG to suppress senescence phenotypes, suggesting the nutrient function of α-KG could be unleashed upon suppression of the epigenetically activated NF-κB in senescence progression (Supplemental Fig. 3H). These findings together suggested that GDH activation could promote α-KG production for efficient removal of H3K9me3 on SASP gene promoters, thus functionally driving SASP induction and aging.

### Manipulations of the FOXQ1-SIRT4-GDH axis affect senescence progression

**Fig. 6:**
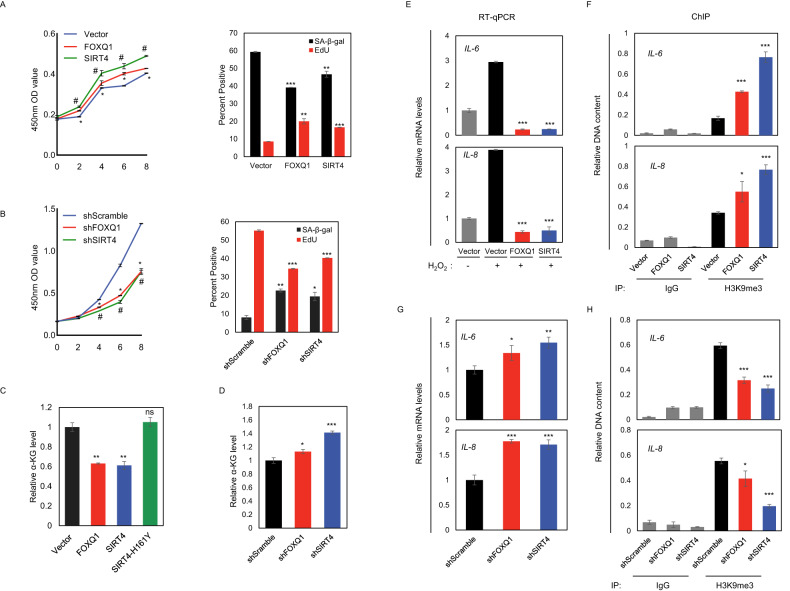
Manipulations of the FOXQ1-SIRT4-GDH axis affect senescence progression. **A** 2BS cells were infected with lentivirus for expressing the indicated genes. Results for the examination of senescence phenotypes by cell proliferation assay, EdU incorporation assay and SA-β-gal staining were shown. Bar represents mean±S.D. for triplicates. **B** 2BS cells were infected with lentivirus for knocking down the indicated genes. Results for the examination of senescence phenotypes by cell proliferation assay, EdU incorporation assay and SA-β-gal staining were shown. Bar represents mean±S.D. for triplicates. **C** Relative α-KG levels were determined in 2BS cells with the indicated overexpression. Bar represents mean±S.D. for triplicates. **D** Relative α-KG levels were determined in 2BS cells with the indicated gene depletion. Bar represents mean±S.D. for triplicates. **E** 2BS cells infected with lentivirus for expressing the indicated genes were further treated by H_2_O_2_. RT-qPCR analysis on the mRNA levels of indicated genes was shown with the bar representing mean±S.D. for triplicates. **F** ChIP analysis on the enrichment pattern of H3K9me3 at *IL-6* and *IL-8* promoters in 2BS cells with the indicated infections. Bar represents mean±S.D. for triplicates. **G** 2BS cells were infected with lentivirus for knocking down the indicated genes. RT-qPCR analysis on the mRNA levels of indicated genes was shown with the bar representing mean±S.D. for triplicates. **H** ChIP analysis on the enrichment pattern of H3K9me3 at *IL-6* and *IL-8* promoters in 2BS cells with the indicated infections. Bar represents mean±S.D. for triplicates. For statistical comparisons in (**A**–**H**), ns, not significant; ^#^*P* < 0.05; ^*^P < 0.05; ^**^P < 0.01; ^***^P < 0.001 (student’s *t* test).

The causal implication of GDH associated α-KG production and epigenetic remodeling at SASP gene loci prompted us to investigate the regulatory role of FOXQ1-SIRT4-GDH axis in senescence. To this end, we constructed both gain-of-function and loss-of-function models in 2BS cells with lentiviral cassettes overexpressing and knocking down FOXQ1 or SIRT4 respectively. Examination of senescence phenotypes by all of the cell proliferation assay, EdU incorporation assay and SA-β-gal staining supported that overexpression of FOXQ1 or SIRT4 could promote cell growth and DNA synthesis, and concordantly inhibited senescence progression partially as indicated by the reduction of SA-β-gal staining compared with control cells (Fig. 6A and Supplemental Fig. 4A). In contrast, depletion of FOXQ1 or SIRT4 suppressed cell growth, and induced premature senescence (Fig. 6B and Supplemental Fig. 4B). Similar results could be observed in IMR90 cell-based senescence model (Supplemental Fig. 4C, D).

Given the negative effect of SIRT4 and FOXQ1 on GDH activities (Figs. 1I and 3D), we speculated that the impact of FOXQ1-SIRT4-GDH axis on cellular senescence might be converged in SASP induction via α-KG dependent mechanism. Indeed, overexpression of FOXQ1 or SIRT4 led to significant decrease of α-KG levels, and this reduction required intact enzymatic function of SIRT4 as the use of catalytic deficient SIRT4 caused no discernible changes (Fig. 6C). Consistently, increased α-KG levels could be detected upon FOXQ1 or SIRT4 depletion (Fig. 6D).

To further consolidate the involvement of α-KG-associated epigenetic reconfiguration at SASP gene loci in senescence, we measured the effect of FOXQ1 and SIRT4 manipulations on the expression of *IL-6* and *IL-8*. The results suggested that overexpression of FOXQ1 or SIRT4 was sufficient to block the induction of *IL-6* and *IL-8* in senescent 2BS cells, while knockdown FOXQ1 or SIRT4 in 2BS showed increased mRNA levels of *IL-6* and *IL-8*, in line with the decreasing trend of FOXQ1 and SIRT4 expressions in senescence (Fig. 6E, G). In agreement with the function of α-KG in histone demethylation, these FOXQ1 or SIRT4 overexpression associated impairment of *IL-6* and *IL-8* production in 2BS correlated with the retention of repressive H3K9me3 on their promoters (Fig. 6F). Conversely, the enrichment of H3K9me3 at *IL-6* and *IL-8* promoters was decreased along with FOXQ1 or SIRT4 depletion (Fig. 6H). Collectively, these data support the model that the FOXQ1-SIRT4-GDH axis plays a critical role in epigenetic control of SASP loci in a GDH and α-KG dependent manner during senescence progression.

Cyclin-dependent kinase inhibitor molecules such as p16 and p21 play critical roles in cellular senescence, so we examined the impact of FOXQ1-SIRT4-GDH axis on the expression of p16 and p21. Western blotting and RT-qPCR analyses suggested that knockdown of FOXQ1 led to decreased p21 expression, while p16 remained unchanged (Supplemental Fig. 4E, F), in line with the transcriptional association between FOXQ1 and p21 in cancer cells (Kaneda et al., 2010a). Meanwhile, SIRT4 and GDH depletion had no significant effect on p21 or p16 expression. We speculate that the long-term impact of FOXQ1/SIRT4/GDH axis on the expression of p16/p21 and other cell cycle inhibitors might be different from the above observed short-term results, as FOXQ1/SIRT4/GDH-associated metabolic regulation might have a gradually accumulated influence.

## Discussion

Identification of novel metabolic regulators implicated in cellular senescence is not only crucial for our understanding of the molecular mechanisms of aging, due to the substantially altered metabolic patterns along with loss of fitness as chronological age advances, but also beneficial to the development of actionable drugs to extend lifespan [28]. In this study, we demonstrated a novel FOXQ1-SIRT4-GDH axis in the control of glutamate dehydrogenase catalyzed production of α-ketoglutaric acid in senescence and aging. We showed the increase of GDH activities in aged cells was linked to its decreased ADP-ribosylation but not to generally altered expression levels, consistent with previous reports that SIRT4-mediated ADP-ribosylation could directly inhibit the enzymatic activity of GDH [18]. Interestingly, we found this post-translational regulation of GDH was associated with loss of FOXQ1-maintained SIRT4 transcription in senescence. More importantly, the shift of balanced FOXQ1-SIRT4-GDH axis in juvenile cells towards a GDH potentiation state in senescence would lead to increased α-KG production and its subsequent intertwining with other hallmarks of aging through epigenetic mechanisms. Indeed, we gave evidence that this induced α-KG production underlies the elevation of histone demethylation against repressive H3K9me3 modification on key secretory and inflammatory gene promoters, thus establishing a novel synergy between altered metabolism and cellular communication in aging. Along with the mechanistic findings, phenotypic examinations suggested that utilization of GDH or α-KG inhibitors would attenuate SASP and delay senescence progression, thus supporting the potential usage of these inhibitors in preventing premature senescence and/or extending healthy lifespan, with additional benefit from the reduced risk of obesity [29] and metabolic syndrome [30, 31], while in awareness of the increased schizophrenia susceptibility [32].

Several members of forkhead family transcription factors, characterized by a common forkhead box (FOX) DNA binding domain [33], have been reported to be involved in metabolism regulation and metabolic diseases [34, 35]. Our identification of FOXQ1 in the maintenance of GDH-catalyzed α-KG production and homeostasis thus supplemented a new player in the list of forkhead TFs with regulatory functions in metabolism [36]. Indeed, FOXQ1 has been found to be overexpressed in variety of human cancers in digestive organs, such as liver, pancreas, and gastrointestinal tracts, where it could enhance tumorigenicity by inducing epithelial-mesenchymal transition, regulating cell cycle, and promoting cell proliferation [37]. The findings of FOXQ1-SIRT4-GDH axis in the control of glutamate dehydrogenase mediated α-ketoglutaric acid production in senescence and aging in this study thus point to possible metabolic abnormalities associated with FOXQ1 overexpression in cancers.

The sirtuin family of nicotinamide adenine dinucleotide (NAD^+^) -dependent deacetylases possess evolutionarily conserved functionalities in metabolism, stress response, genomic stability, and longevity [38, 39]. In fact, due to the roles of NAD^+^ as a coenzyme in redox reactions and in regulating NAD^+^-consuming enzyme activities [40], nutrient and energy homeostasis in shaping the aging process and determining lifespan in diverse organisms was largely attributed to the regulated NAD^+^-dependent redox reactions, deacetylation (at histone tails and non-histone proteins), and communications between nucleus and mitochondria [41]. Indeed, decreased NAD^+^ levels have been observed as age advances, and this change could drive global epigenetic remodeling with deregulated histone acetylation levels to influence aging-associated pathologies [40]. Here, we showed that in addition to the well-recognized roles of NAD^+^, loss of SIRT4, one of the less characterized sirtuins, could accelerate cellular senescence and aging in a α-KG-coordinated manner. These results agreed with a previous study focused on *Drosophila melanogaster* Sirt4, which participated in metabolic response to fasting and in lifespan extension [42].

Functional importance of α-KG for histone demethylases and TET family DNA demethylases [43] in epigenetic regulation has been largely demonstrated in human malignancies driven by genetic mutations of IDH1/2 enzymes, which promote overproduction of 2HG – structural analogue but functional inhibitor of α-KG – and lead to profound epigenetic alterations such as aberrant histone and/or DNA hyper-methylations in cancers [27]. Our data suggest that GDH and α-KG enhanced removal of H3K9me3 on key inflammation gene promoters was critical for SASP induction in senescence, consistent with the general tendency of loss of H3K9me3 represented heterochromatin in aged cells [44]. Despite the multiple facets of SASP factors in senescent phenotypes, deteriorating roles of SASP, such as promotion of persistent chronic inflammation, would contribute to a variety of age-related pathologies including impaired tissue structure and tumor development, where it might stimulate neoplastic cell growth, tumor angiogenesis, and metastasis, thereby invoking the development of late life cancers [45].

## Experimental procedures

### Cell culture and animal experiments

Human embryonic lung diploid fibroblast 2BS cells (National Institute of Biological Products, Beijing, China) were cultured in RPMI 1640 medium (GIBCO) supplemented with 10% fetal bovine serum (FBS, GIBCO) and human cell lines HEK293, HEK293T were cultured in Dulbecco’s modified Eagle’s medium (DMEM) supplemented with 10% FBS (HyClone) and 100 U/ml penicillin and 100 µg/ml streptomycin, and the cells were then cultured at 37 °C in 5% CO_2_. Replicative senescence in 2BS cells was attained after 55 passages in culture while 2BS cells before PD 30 were considered young. For the induction of premature senescence, 2BS cells at about 50% confluence were briefly exposed to 200 μM H_2_O_2_ for 2 h. The cells were washed twice with DMEM to remove the residual H_2_O_2_, re-cultured in fresh complete medium, and subjected to the subsequent tests. For oncogene-induced senescence, IMR90 cells were transduced with an ER:RAS (kindly provided by Dr. Masashi Narita, Cancer Research U.K., Cambridge Research Institute) and given 100 nM 4-hydroxytamoxifen (4-OHT) to induce ER-RAS fusion protein expression and maintained in 4-OHT–containing DMEM (w/o phenol red) with 10% FBS until harvesting. Rapamycin (Selleck Chemicals, S1039) was added 12 h before harvest at a final concentration of 100 nM. 2BS cells were exposed to Octyl-(R)-2HG (Sigma, SML2200) which is cell-permeable at a final concentration of 500 μM for 24 h. R162 and EGCG were purchased from MedChem Express (MCE, USA). BAY 11-7082 was purchased from Selleck Chemicals. BALB/c and ICR mice were maintained in a certified animal facility in accordance with the guidelines set forth by the Peking University Animal Ethics Committee. Five or six animals per group were used with no randomization or blinding. For aging studies, livers and brains of young (3 months) or old (24 to 28 months) BALB/c mice (male) were collected for analysis. For aging studies, livers and brains of young (3 months) or old (24 to 28 months) BALB/c mice (male) were collected for analysis. To test the effect of EGCG on the in vivo α-KG production and SASP expression, 12-month-old ICR mice (male) were orally treated with EGCG for 1 month, and then serums were extracted for ELISA assays (Servicebio, GEM0001); the brain and liver tissues were isolated for α-KG assay and western blotting analyses.

### Statistical analysis

Statistical results were obtained using Student’s *t* test or ANOVA. *P* < 0.05 was considered statistically significant.

## Supplementary information

Reproducibility checklist

Supplemental figures

Supplemental text

Original Data File

## Data Availability

Data sharing is not applicable to this article as no new data were created or analyzed in this study.

## References

[1] Lopez-Otin C, Blasco MA, Partridge L, Serrano M, Kroemer G (2013). The hallmarks of aging. Cell.

[2] Kaelin WG, McKnight SL (2013). Influence of metabolism on epigenetics and disease. Cell..

[3] Detich N, Hamm S, Just G, Knox JD, Szyf M (2003). The methyl donor S-Adenosylmethionine inhibits active demethylation of DNA: a candidate novel mechanism for the pharmacological effects of S-Adenosylmethionine. J Biol Chem.

[4] Hausinger RP (2004). Fe(II)/alpha-ketoglutarate-dependent hydroxylases and related enzymes. Crit Rev Biochem Mol.

[5] Yan H, Parsons DW, Jin G, McLendon R, Rasheed BA, Yuan W (2009). IDH1 and IDH2 mutations in gliomas. N. Engl J Med.

[6] Figueroa ME, Abdel-Wahab O, Lu C, Ward PS, Patel J, Shih A (2010). Leukemic IDH1 and IDH2 mutations result in a hypermethylation phenotype, disrupt TET2 function, and impair hematopoietic differentiation. Cancer Cell.

[7] Plaitakis A, Kalef-Ezra E, Kotzamani D, Zaganas I, Spanaki C (2017). The Glutamate Dehydrogenase Pathway and Its Roles in Cell and Tissue Biology in Health and Disease. Biology..

[8] McEntee WJ, Crook TH (1993). Glutamate: its role in learning, memory, and the aging brain. Psychopharmacology..

[9] Bao XD, Pal R, Hascup KN, Wang YF, Wang WT, Xu WH (2009). Transgenic Expression of Glud1 (Glutamate Dehydrogenase 1) in Neurons: In Vivo Model of Enhanced Glutamate Release, Altered Synaptic Plasticity, and Selective Neuronal Vulnerability. J Neurosci.

[10] Kuilman T, Michaloglou C, Vredeveld LCW, Douma S, van Doom R, Desmet CJ (2008). Oncogene-induced senescence relayed by an interleukin-dependent inflammatory network. Cell.

[11] McHugh D, Gil J (2018). Senescence and aging: Causes, consequences, and therapeutic avenues. J Cell Biol.

[12] Chien Y, Scuoppo C, Wang X, Fang X, Balgley B, Bolden JE (2011). Control of the senescence-associated secretory phenotype by NF-κB promotes senescence and enhances chemosensitivity. Genes Dev.

[13] Bhatt D, Ghosh S (2014). Regulation of the NF-κB-Mediated Transcription of Inflammatory Genes. Front Immunol.

[14] Wu N, Yang M, Gaur U, Xu H, Yao Y, Li D (2016). Alpha-Ketoglutarate: Physiological Functions and Applications. Biomol Ther (Seoul).

[15] Lopez-Lluch G, Hunt N, Jones B, Zhu M, Jamieson H, Hilmer S (2006). Calorie restriction induces mitochondrial biogenesis and bioenergetic efficiency. Proc Natl Acad Sci.

[16] Botman D, Tigchelaar W, Van, Noorden CJF (2014). Determination of Glutamate Dehydrogenase Activity and Its Kinetics in Mouse Tissues using Metabolic Mapping (Quantitative Enzyme Histochemistry). J Histochem Cytochem.

[17] Andersen JV, Jakobsen E, Waagepetersen HS, Aldana BI (2019). Distinct differences in rates of oxygen consumption and ATP synthesis of regionally isolated non-synaptic mouse brain mitochondria. J Neurosci Res.

[18] Haigis MC, Mostoslavsky R, Haigis KM, Fahie K, Christodoulou DC, Murphy AJ (2006). SIRT4 inhibits glutamate dehydrogenase and opposes the effects of calorie restriction in pancreatic beta cells. Cell..

[19] Ahuja N, Schwer B, Carobbio S, Waltregny D, North BJ, Castronovo V (2007). Regulation of insulin secretion by SIRT4, a mitochondrial ADP-ribosyltransferase. J Biol Chem.

[20] Herrero-Yraola A, Bakhit SM, Franke P, Weise C, Schweiger M, Jorcke D (2001). Regulation of glutamate dehydrogenase by reversible ADP-ribosylation in mitochondria. Embo J.

[21] Csibi A, Fendt SM, Li C, Poulogiannis G, Choo AY, Chapski DJ (2013). The mTORC1 pathway stimulates glutamine metabolism and cell proliferation by repressing SIRT4. Cell..

[22] Yan J, Enge M, Whitington T, Dave K, Liu JP, Sur I (2013). Transcription Factor Binding in Human Cells Occurs in Dense Clusters Formed around Cohesin Anchor Sites. Cell..

[23] Li Q, Zhang Y, Fu J, Han L, Xue L, Lv C (2013). FOXA1 mediates p16(INK4a) activation during cellular senescence. EMBO J.

[24] Acosta JC, O'Loghlen A, Banito A, Guijarro MV, Augert A, Raguz S (2008). Chemokine signaling via the CXCR2 receptor reinforces senescence. Cell.

[25] Jin L, Li D, Alesi GN, Fan J, Kang HB, Lu Z (2015). Glutamate dehydrogenase 1 signals through antioxidant glutathione peroxidase 1 to regulate redox homeostasis and tumor growth. Cancer Cell.

[26] Li C, Allen A, Kwagh J, Doliba NM, Qin W, Najafi H (2006). Green tea polyphenols modulate insulin secretion by inhibiting glutamate dehydrogenase. J Biol Chem.

[27] Xu W, Yang H, Liu Y, Yang Y, Wang P, Kim SH (2011). Oncometabolite 2-Hydroxyglutarate Is a Competitive Inhibitor of alpha-Ketoglutarate-Dependent Dioxygenases. Cancer Cell.

[28] Lopez-Otin C, Galluzzi L, Freije JMP, Madeo F, Kroemer G (2016). Metabolic Control of Longevity. Cell..

[29] Vetterli L, Carobbio S, Frigerio F, Karaca M, Maechler P (2016). The Amplifying Pathway of the beta-Cell Contributes to Diet-induced Obesity. J Biol Chem.

[30] Ortsater H, Grankvist N, Wolfram S, Kuehn N, Sjoholm A (2012). Diet supplementation with green tea extract epigallocatechin gallate prevents progression to glucose intolerance in db/db mice. Nutr Metab (Lond).

[31] Pournourmohammadi S, Grimaldi M, Stridh MH, Lavallard V, Waagepetersen HS, Wollheim CB (2017). Epigallocatechin-3-gallate (EGCG) activates AMPK through the inhibition of glutamate dehydrogenase in muscle and pancreatic ss-cells: A potential beneficial effect in the pre-diabetic state?. Int J Biochem Cell Biol.

[32] Lander SS, Khan U, Lewandowski N, Chakraborty D, Provenzano FA, Mingote S (2019). Glutamate Dehydrogenase-Deficient Mice Display Schizophrenia-Like Behavioral Abnormalities and CA1-Specific Hippocampal Dysfunction. Schizophr Bull.

[33] Weigel D, Jackle H (1990). The Fork Head Domain - a Novel DNA-Binding Motif of Eukaryotic Transcription Factors. Cell..

[34] Greer EL, Brunet A (2008). FOXO transcription factors in ageing and cancer. Acta Physiol (Oxf).

[35] Masui K, Tanaka K, Akhavan D, Babic I, Gini B, Matsutani T (2013). mTOR complex 2 controls glycolytic metabolism in glioblastoma through FoxO acetylation and upregulation of c-Myc. Cell Metab.

[36] Carlsson P, Mahlapuu M (2002). Forkhead transcription factors: Key players in development and metabolism. Dev Biol.

[37] Kaneda H, Arao T, Tanaka K, Tamura D, Aomatsu K, Kudo K (2010). FOXQ1 Is Overexpressed in Colorectal Cancer and Enhances Tumorigenicity and Tumor Growth. Cancer Res.

[38] Haigis MC, Sinclair DA (2010). Mammalian sirtuins: biological insights and disease relevance. Annu Rev Pathol.

[39] Sebastian C, Satterstrom FK, Haigis MC, Mostoslavsky R (2012). From sirtuin biology to human diseases: an update. J Biol Chem.

[40] Imai S, Guarente L (2014). NAD+ and sirtuins in aging and disease. Trends Cell Biol.

[41] Imai SI, Guarente L (2016). It takes two to tango: NAD(+) and sirtuins in aging/longevity control. NPJ Aging Mech Dis.

[42] Wood JG, Schwer B, Wickremesinghe PC, Hartnett DA, Burhenn L, Garcia M (2018). Sirt4 is a mitochondrial regulator of metabolism and lifespan in Drosophila melanogaster. P Natl Acad Sci.

[43] Zdzisinska B, Zurek A, Kandefer-Szerszen M (2017). Alpha-Ketoglutarate as a Molecule with Pleiotropic Activity: Well-Known and Novel Possibilities of Therapeutic Use. Arch Immunol Ther Ex.

[44] Benayoun BA, Pollina EA, Brunet A (2015). Epigenetic regulation of ageing: linking environmental inputs to genomic stability. Nat Rev Mol Cell Biol.

[45] Coppe JP, Desprez PY, Krtolica A, Campisi J (2010). The Senescence-Associated Secretory Phenotype: The Dark Side of Tumor Suppression. Annu Rev Pathol-Mech.

