# Supplementary material for: Epigenetic activation of secretory phenotypes in senescence by the FOXQ1-SIRT4-GDH signaling
Source: Cell Death Dis. 2023 Jul 29;14(7):481. doi: 10.1038/s41419-023-06002-9 (PMC10387070; doi:10.1038/s41419-023-06002-9)
Supplement: Supplementary file 2 — Supplemental figures [file 41419_2023_6002_MOESM2_ESM.pdf]

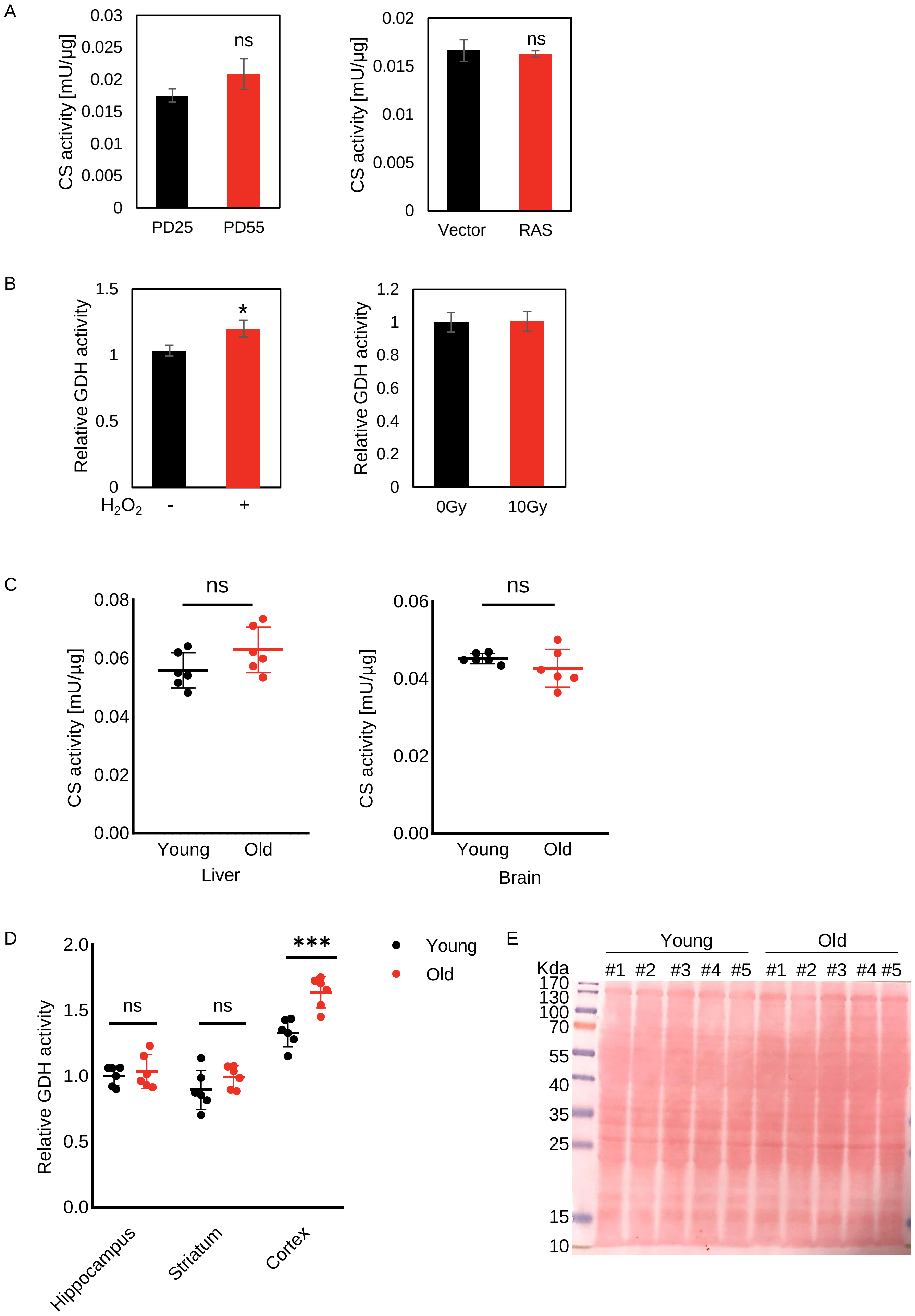
A
CS activity [mU/μg]
0.03
0.025
0.02
0.015
0.01
0.005
0
ns
PD25
PD55
0.02
0.015
0.01
0.005
0
ns
Vector
RAS
B
Relative GDH activity
1.5
1
0.5
0
*
H_2O_2
-
+
1.2
1
0.8
0.6
0.4
0.2
0
0Gy
10Gy
C
0.08
0.06
0.04
0.02
0.00
ns
Young
Old
Liver
0.06
0.04
0.02
0.00
ns
Young
Old
Brain
D
2.0
1.5
1.0
0.5
0.0
ns
ns
***
Hippocampus
Striatum
Cortex
Young
Old
E
Young
Old
Kda
#1 #2 #3 #4 #5 #1 #2 #3 #4 #5
170
130
100
70
55
40
35
25
15
10

Supplemental Figure 1

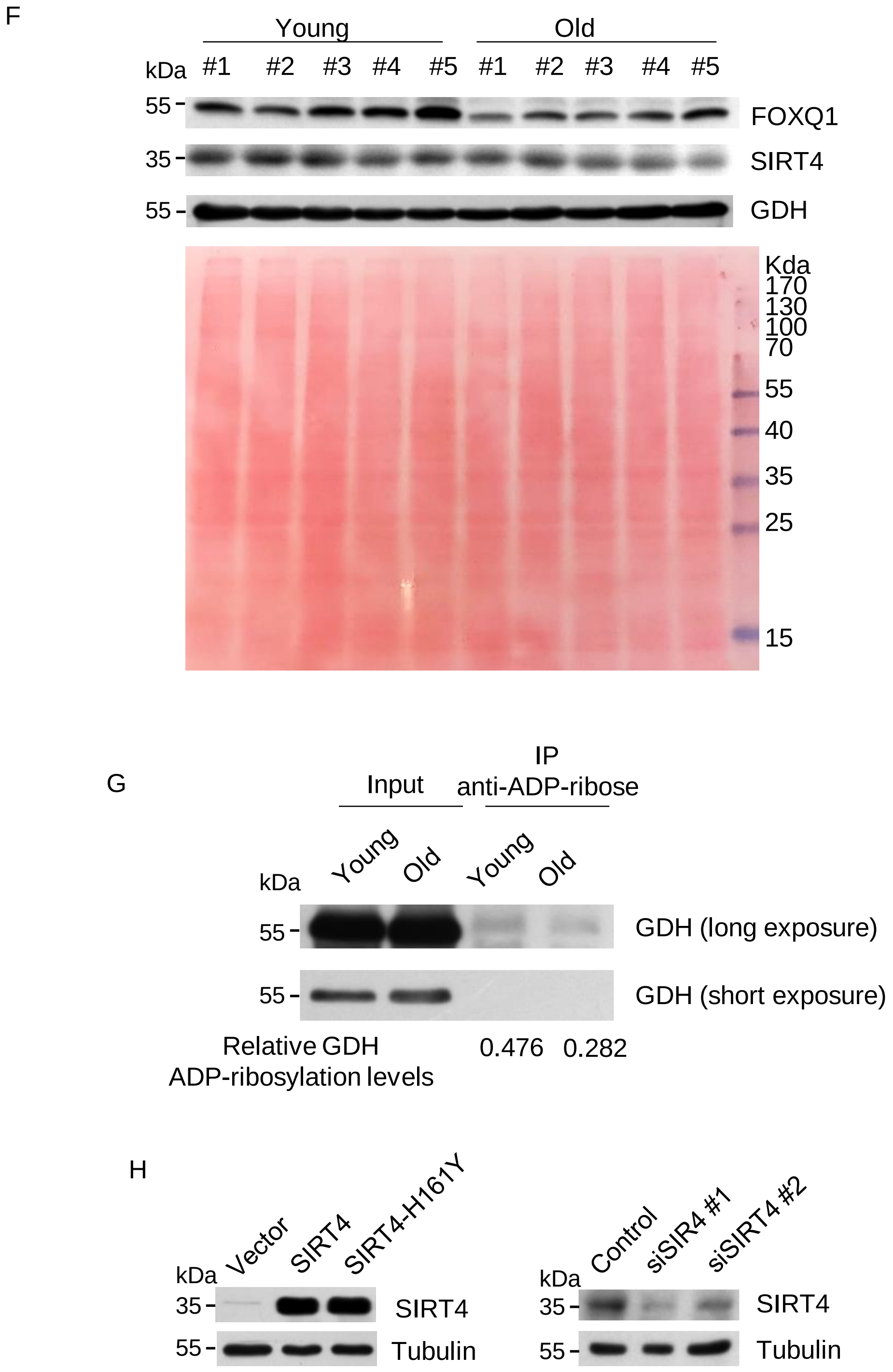

Supplemental Figure 1. Glutamate Dehydrogenase Activity Is Increased in Aging and Cellular Senescence (A) CS activity of replicative senescent 2BS cells and RAS-induced senescent IMR90 cells. (B) Relative GDH activity was determined in H_2O_2-induced and IR-induced premature senescent 2BS cells. (C) CS activity was determined in liver and brain tissues of young (3 months) and old BALB/c mice (26 months). Each point in the scatter plot represents a mouse, and the line represents group means ± S.D. (n = 6). (D) GDH activity analysis of cortex, hippocampus, and striatum regions of young (3 months) and old (26 months) BALB/c mice brain. Each point in the scatter plot represents a mouse, and the line represents group means ± S.D. (n = 6). (E) Ponceau stained membrane for the blot presented in Figure 1D. (F) Levels of indicated protein in livers of young (3 months) and old (26 months) BALB/c mice (top). Ponceau staining of the membrane is shown as loading control (bottom). (G) Immunoprecipitation in brains of young and old mice was performed with the use of anti-ADP-ribose antibody. GDH proteins with ADP-ribosylation modification were then probed by anti-GDH antibody conjugated with TrueBlot® Secondary Antibodies (HRP). GDH ADP-ribosylation levels indicated below were quantified as pull-downed band density relative to the relevant input. (H) Protein levels of SIRT4 in HEK293 cells with indicated transfections. For statistical comparisons, *P < 0.05; **P < 0.01; ***P < 0.001; ns, not significant (Student's t test).

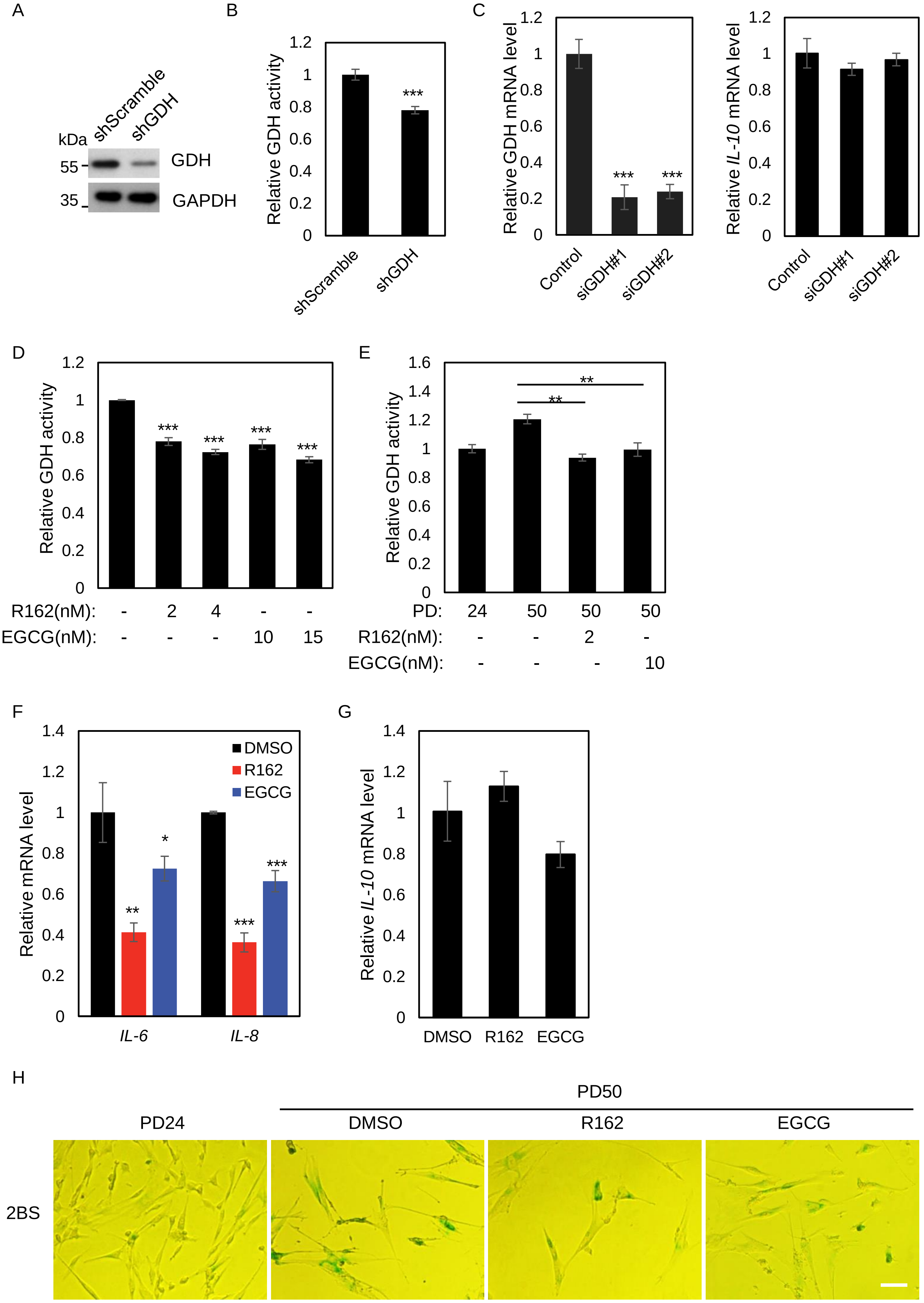
A
kDa
shScramble
shGDH
55
35
GDH
GAPDH
B
Relative GDH activity
***
shScramble
shGDH
C
Relative GDH mRNA level
***
***
Control
siGDH#1
siGDH#2
Relative IL-10 mRNA level
D
Relative GDH activity
R162(nM): - 2 4 - -
EGCG(nM): - - - 10 15
E
Relative GDH activity
**
**
PD: 24 50 50 50
R162(nM): - - 2 -
EGCG(nM): - - - 10
F
Relative mRNA level
DMSO
R162
EGCG
IL-6
IL-8
G
Relative IL-10 mRNA level
DMSO
R162
EGCG
H
PD50
PD24
DMSO
R162
EGCG
2BS

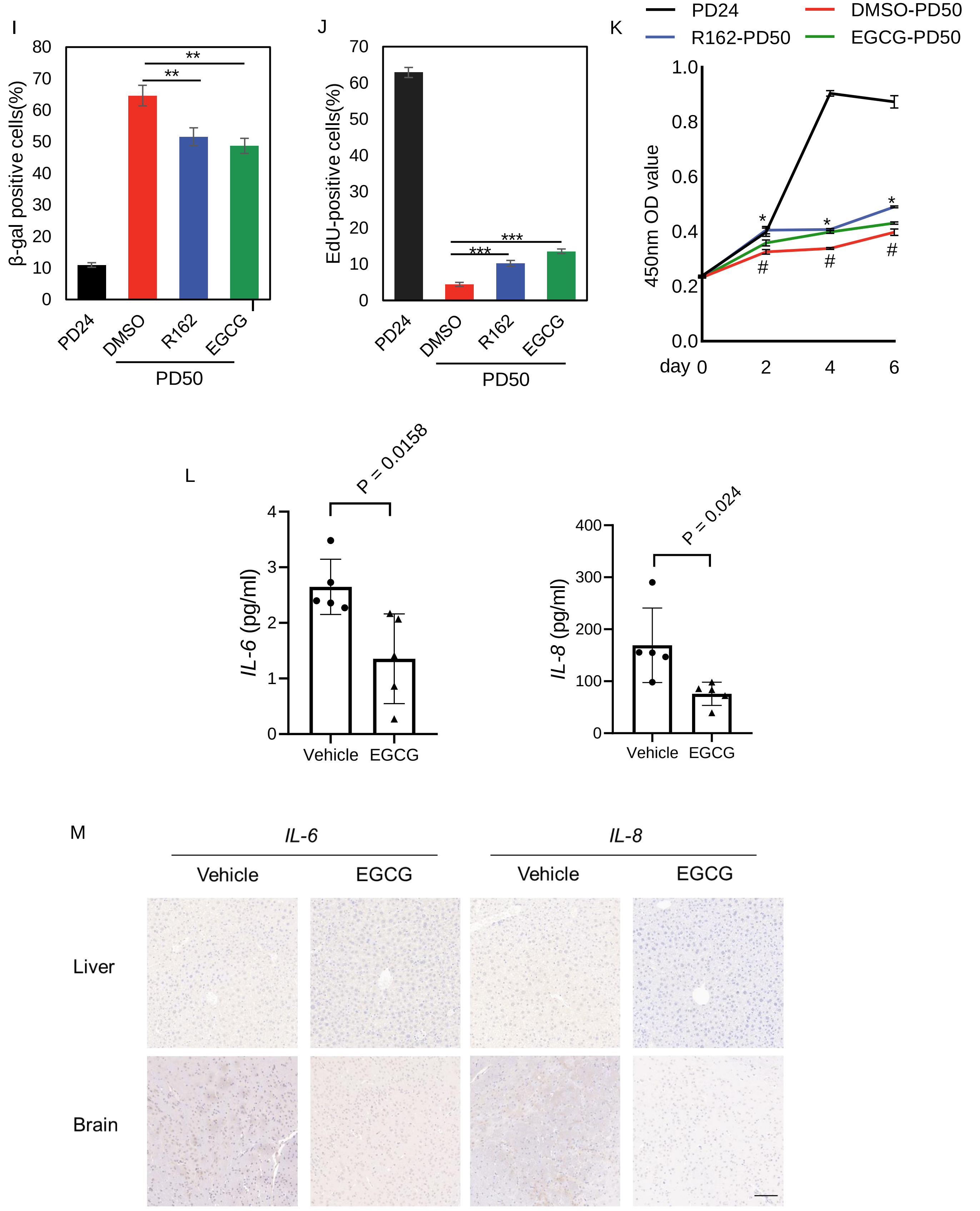
I
β-gal positive cells(%)
80
70
60
50
40
30
20
10
0
**
**
PD24
DMSO
R162
EGCG
PD50
J
EdU-positive cells(%)
70
60
50
40
30
20
10
0
***
***
PD24
DMSO
R162
EGCG
PD50
K
PD24
R162-PD50
DMSO-PD50
EGCG-PD50
450nm OD value
1.0
0.8
0.6
0.4
0.2
0.0
day 0
2
4
6
*
#
L
IL-6 (pg/ml)
P = 0.0158
4
3
2
1
0
Vehicle
EGCG
IL-8 (pg/ml)
P = 0.024
400
300
200
100
0
Vehicle
EGCG
M
IL-6
IL-8
Vehicle
EGCG
Vehicle
EGCG
Liver
Brain

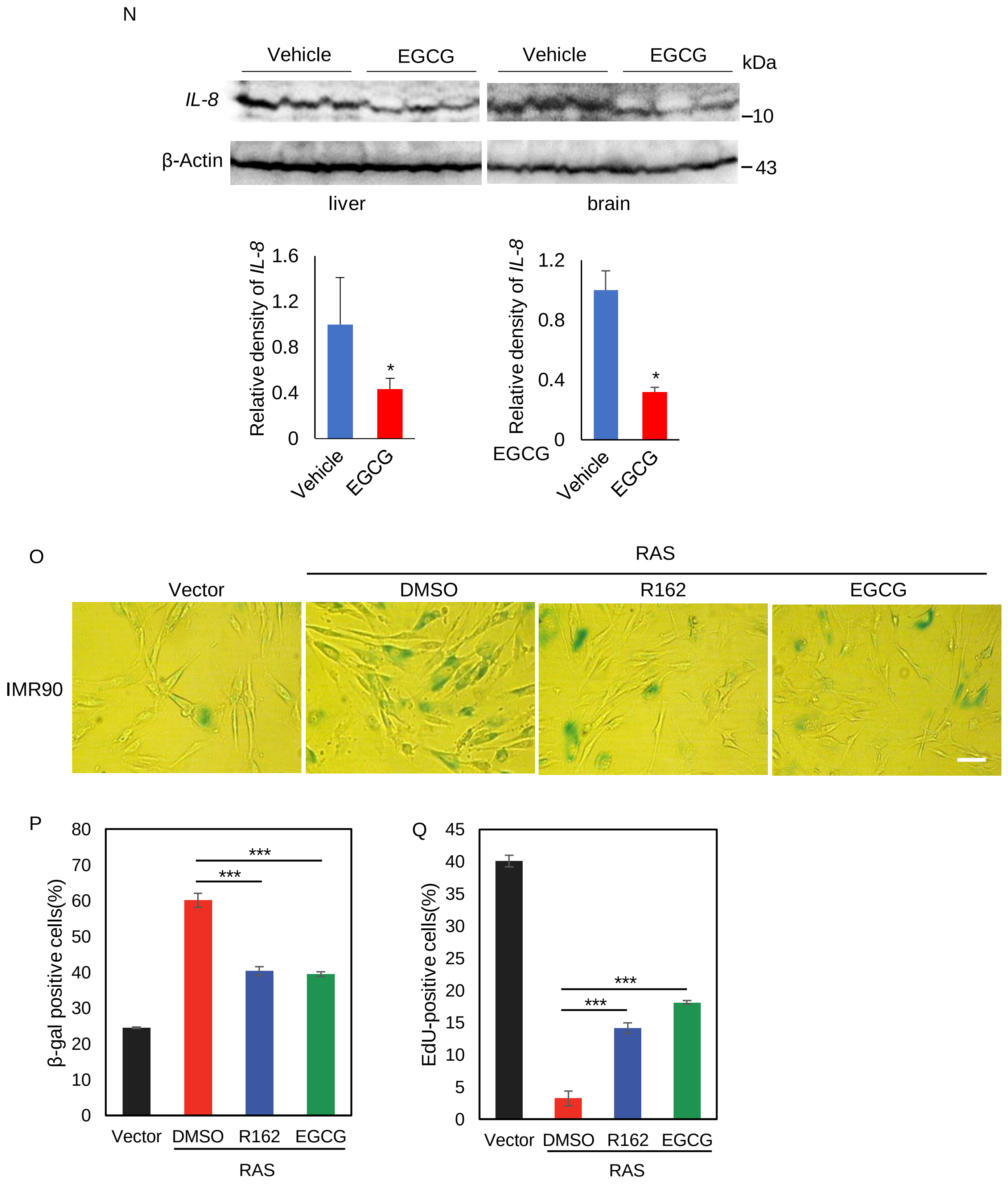
N
Vehicle
EGCG
Vehicle
EGCG
kDa
IL-8
−10
β-Actin
−43
liver
brain
Relative density of IL-8
1.6
1.2
0.8
0.4
0
*
Vehicle
EGCG
Relative density of IL-8
1.2
0.8
0.4
0
EGCG
*
Vehicle
EGCG
O
RAS
Vector
DMSO
R162
EGCG
IMR90
P
β-gal positive cells(%)
80
70
60
50
40
30
20
10
0
***
***
Vector
DMSO
R162
EGCG
RAS
Q
EdU-positive cells(%)
45
40
35
30
25
20
15
10
5
0
***
***
Vector
DMSO
R162
EGCG
RAS

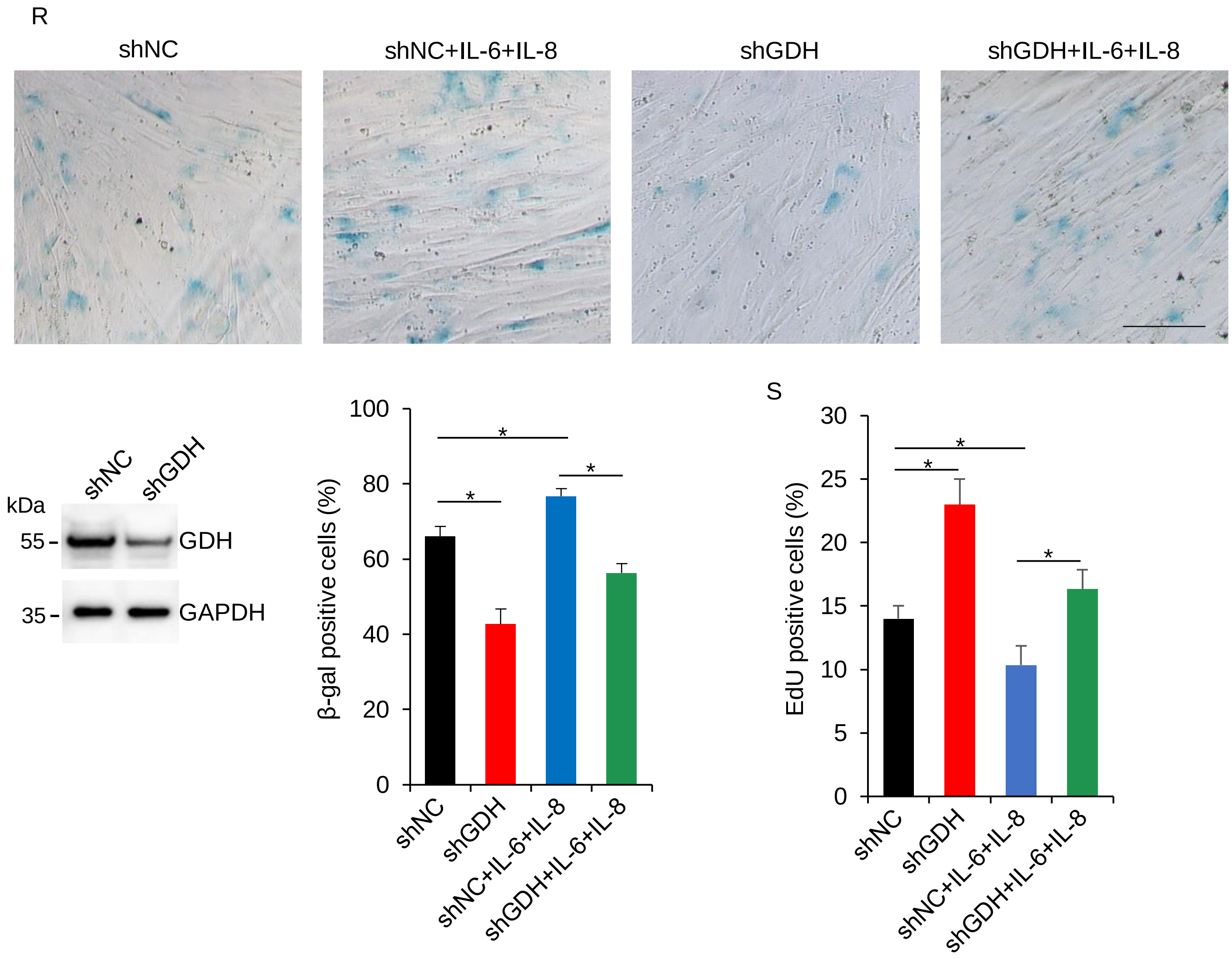

Supplemental Figure 2. GDH inhibition Attenuates the Secretory Phenotypes in Senescence Progression. (A) GDH knockdown efficiency in senescent 2BS cells was validated by western blotting analysis. (B) Relative GDH activity in GDH stably depleted 2BS cells as in (A) was measured. Bar represents mean ± S.D. for triplicates. (C) Relative mRNA levels of indicated genes in senescent 2BS cells transfected with indicated siRNAs. Bar represents mean ± S.D. for triplicates. (D-E) Relative GDH activity in 2BS cells with the indicated PDs and drugs. Bar represents mean ± S.D. for triplicates. (F-G) RT-qPCR analysis on the mRNA levels of indicated genes in 2BS cells with the indicated treatment. Bar represents mean ± S.D. for triplicates. (H-I) SA-β-gal analysis in 2BS cells with the indicated PDs and drug treatment. Representative immunofluorescence images were shown in (H). Scale bar, 100 µm. Percentage of cells positive for SA-β-gal staining was shown in (I). Bar represents mean ± S.D. for triplicates. (J) EdU incorporation was performed in 2BS cells with the indicated treatment. Bar represents mean ± S.D. for triplicates. (K) Growth curves of 2BS cells treated with DMSO (control), R162 (2 nM) or EGCG (10 nM) were determined by CCK-8 assay. Data were represented as the mean ± S.D. for triplicates. *P < 0.05, R162 versus DMSO; #P < 0.05, EGCG versus DMSO. (L-N) 12-momth old ICR mice were treated with or without EGCG for 1 month. ELISA assays were performed to determine the *IL-6* and *IL-8* levels in serum (L). (n=5) immunohistochemistry analysis of *IL-6* and *IL-8* levels in the liver and brain tissues (M). Scale bar, 100 µm. Western blotting analysis of *IL-8* expression changes in the liver and brain tissues (N) *P < 0.05. (O) SA-β-gal analysis was performed in RAS-induced senescence model in IMR90 cells with the indicated treatment. Representative immunofluorescence images were shown. Scale bar, 100 µm. (P) Percentage of cells positive for SA-β-gal staining was shown. Bar represents mean ± S.D. for triplicates. (Q) EdU incorporation was performed in IMR90 cells with the indicated treatment. Bar represents mean ± S.D. for triplicates. *P<0.05; **P < 0.01; ***P < 0.001. (R-S) GDH stably depleted or control 2BS cells were cultured in the presence of *IL-6* and *IL-8* (50ng/ml) for 1 month followed by SA-β-gal staining (R) and EdU incorporation assays (S). Scale bar, 100 µm. Data were represented as the mean ± S.D. for triplicates. *P < 0.05, two-way ANOVA. For statistical analysis in (B, L, N), student's t test was used; for (C-G, I-K, P, Q), one-way ANOVA was used.

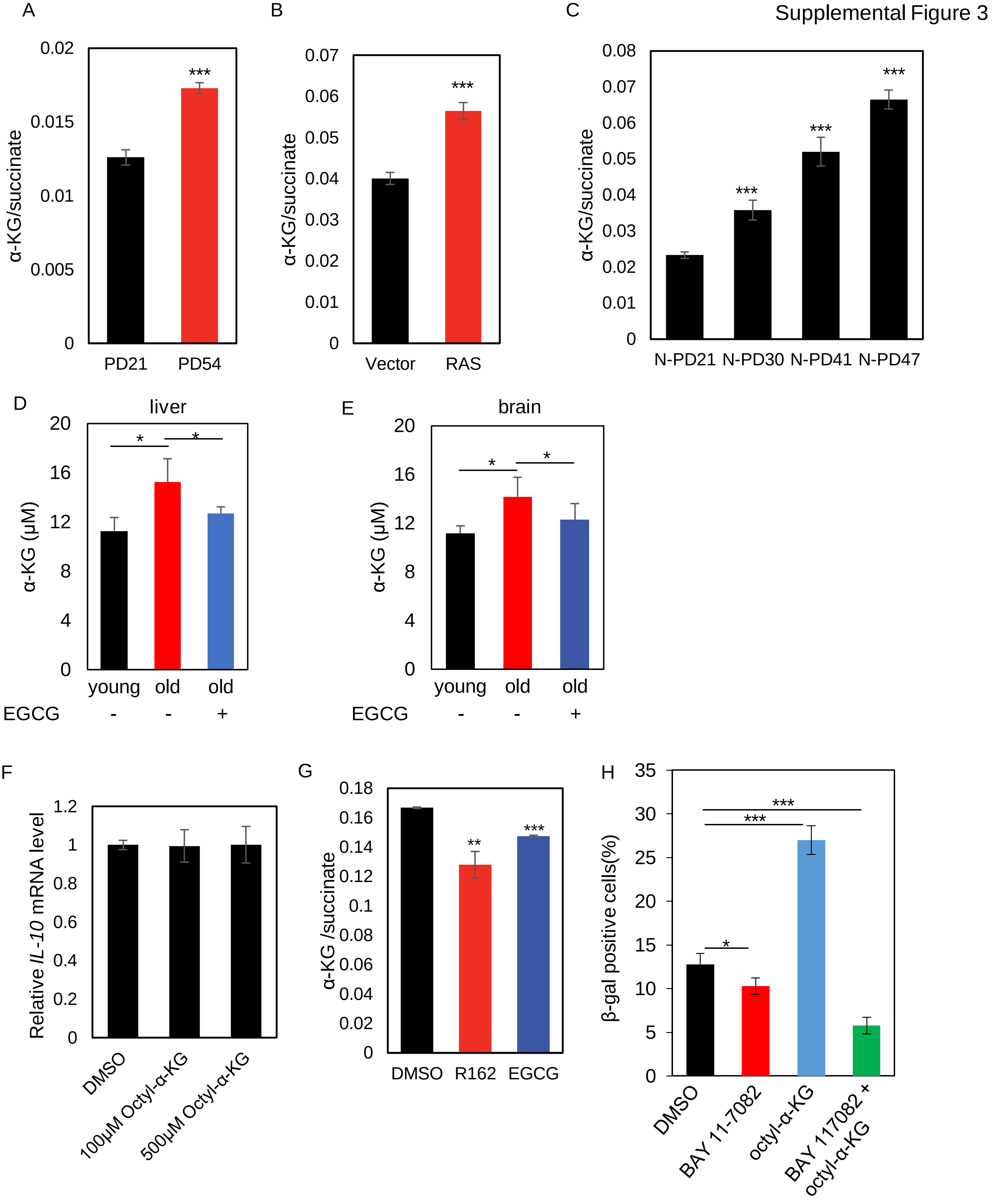

Supplemental Figure 3. Increased α-ketoglutarate Production Potentiates Histone/DNA Demethylation at Key Inflammatory Gene Promoters in Senescence. (A) Ratio of intracellular αKG/succinate concentration in 2BS cells at the indicated PDs. Bar represents mean ± S.D. for triplicates. (B) Ratio of intracellular αKG/succinate concentration in RAS-induced senescence model in IMR90 cells. Bar represents mean ± S.D. for triplicates. (C) Ratio of nuclear αKG/succinate concentration were determined by using isolated nuclei of 2BS cells at the indicated PDs. Bar represents mean ± S.D. for triplicates. (D-E) 12-month old ICR mice were treated with or without EGCG for 1 month followed by a-KG assays using liver and brain tissues compared with the 4-week old young mice. (n=5, two-way ANOVA)
(F) RT-qPCR analysis on *IL-10* mRNA levels in 2BS cells with the indicated treatment. Bar represents mean ± S.D. for triplicates. (G) Ratio of intracellular αKG/succinate concentration in 2BS cells with the indicated treatment. Bar represents mean ± S.D. for triplicates. (H) SA-β-gal analysis was performed in 2BS cells with the indicated treatment. Percentage of cells positive for SA-β-gal staining was shown. Bar represents mean ± S.D. for triplicates (one-way ANOVA). Student's t-test was used for statistical comparisons in (A-C, F, G). *$P < 0.05$; **$P < 0.01$; ***$P < 0.001$.

Supplemental Figure 4

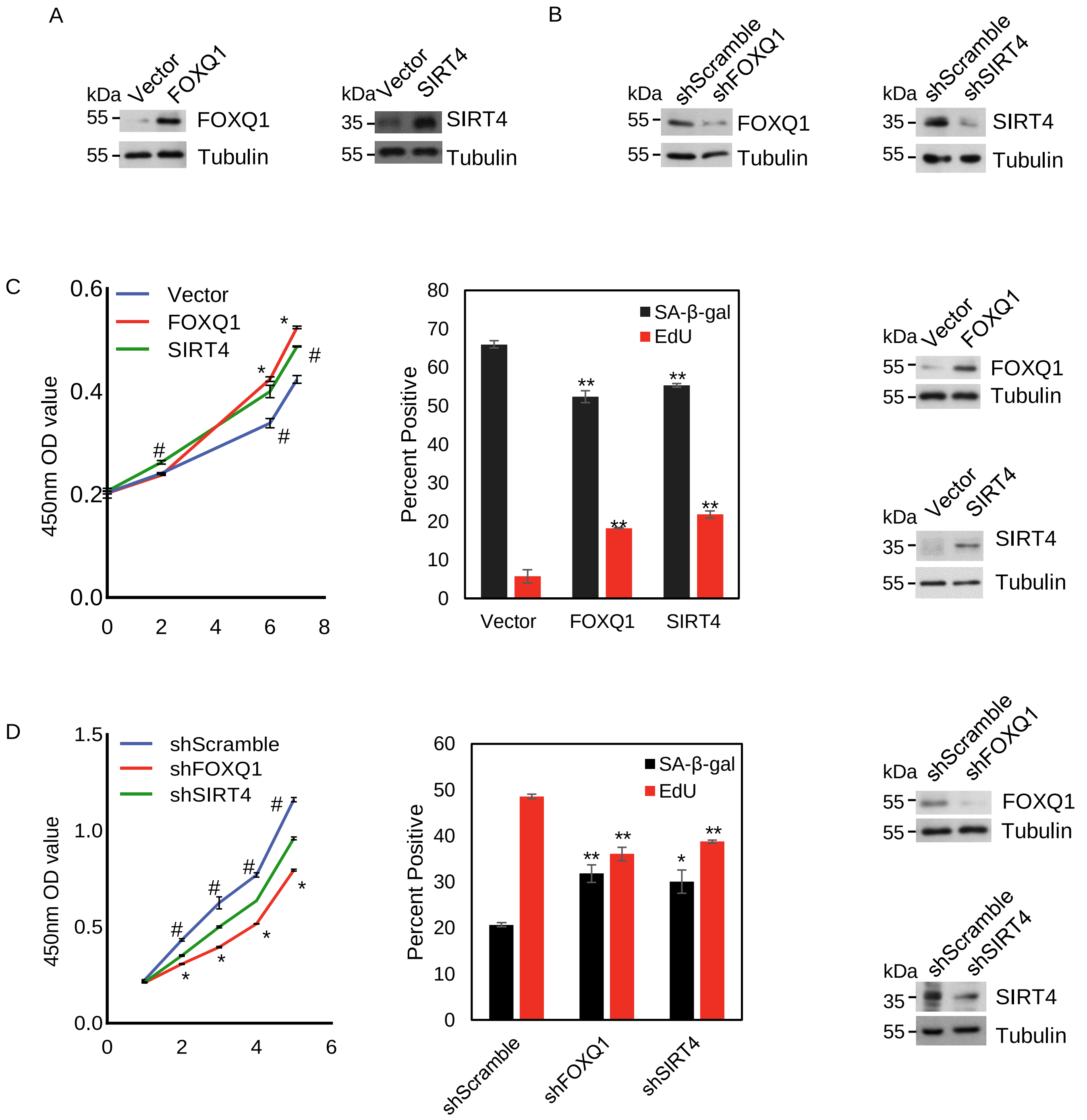

E

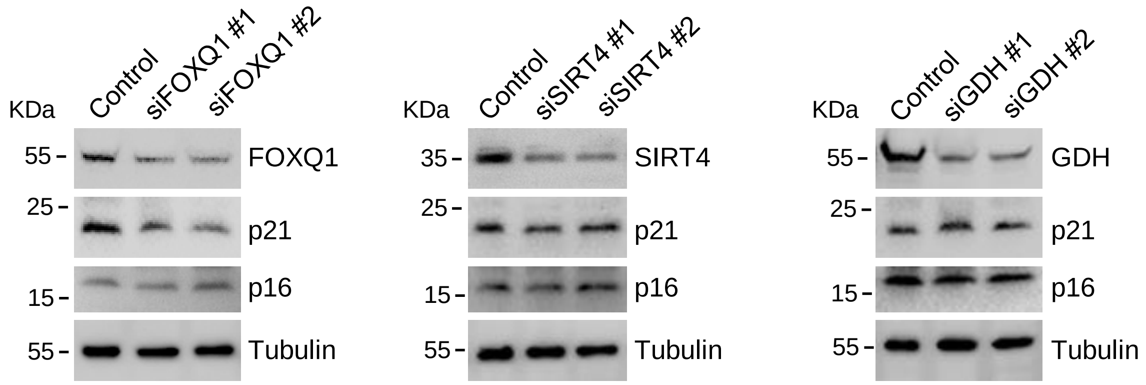

F

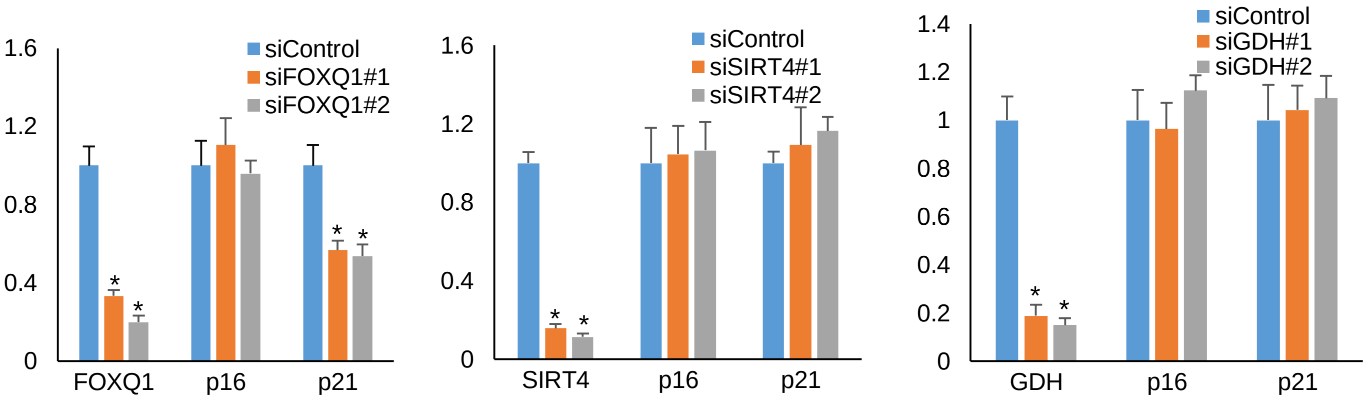

Supplemental Figure 4. Manipulations of the FOXQ1-SIRT4-GDH Axis Affect Senescence Progression. (A) FOXQ1 and SIRT4 protein levels in 2BS cells stably integrated with the indicated constructs for gene expression. (B) FOXQ1 and SIRT4 protein levels in 2BS cells stably integrated with the indicated constructs for RNAi. (C) RAS-induced senescent IMR90 cells were further infected with the indicated lentiviral constructs. Cell proliferation (left), EdU incorporation and SA-β-gal staining results (middle) in these cells were shown. Data were represented as the mean ± S.D. for triplicates in the line plot; *P < 0.05, FOXQ1 versus Vector; #P < 0.05, SIRT4 versus Vector (one-way ANOVA). Bar represents mean ± S.D. for triplicates; *P < 0.05; **P < 0.01. FOXQ1 and SIRT4 protein levels in these cells were shown on right. (D) IMR90 cells (PD24) were infected with the indicated lentiviral constructs for RNAi. Cell proliferation (left), EdU incorporation and SA-β-gal staining results (middle) in these cells were shown. Data were represented as the mean ± S.D. for triplicates in the line plot; *P < 0.001, shFOXQ1 versus shScramble; #P < 0.001, shSIRT4 versus shScramble. Bar represents mean ± S.D. for triplicates; *P < 0.05; **P < 0.01 (one-way ANOVA). FOXQ1 and SIRT4 protein levels in these cells were shown on right. (E-F) 2BS cells were transfected with indicated siRNA. The mRNA or protein level was measured by western blotting (E) or RT-qPCR (F) Bar represents mean ± S.D. for triplicates. *P < 0.05, siRNA versus Control (student's t-test).
