# Supplementary material for: Epigenetic activation of secretory phenotypes in senescence by the FOXQ1-SIRT4-GDH signaling
Source: Cell Death Dis. 2023 Jul 29;14(7):481. doi: 10.1038/s41419-023-06002-9 (PMC10387070; doi:10.1038/s41419-023-06002-9)
Supplement: Supplementary file 3 — Supplemental text [file 41419_2023_6002_MOESM3_ESM.docx]

**SUPPLEMENTAL NOTE**

The contribution of GDH/α-KG to senescence might be twofold. Apart from the role of α-KG as a cofactor in histone/DNA demethylation to modulate senescent epigenome, the function of α-KG as TCA-cycle intermediate (Wu et al., 2016), and glutamine/glutamate-based nitrogen exchange, carbon supplies, redox balance and other biosynthetic pathways (Cruzat, Macedo Rogero, Noel Keane, Curi, & Newsholme, 2018) shall be investigated to fully evaluate the influence of increased GDH activities in senescence. Indeed, inhibition of α-KG fostered SASP by blocking NF-κB attenuated but not accelerated the senescence progression, suggesting the beneficial effect of GDH/α-KG could be unleashed upon suppression of the epigenetically activated NF-κB in senescence. In this regard, GDH as a key enzyme in glutaminolysis for the conversion of glutamine into TCA cycle metabolites might support the utilization of glutamine/glutamate as carbon donor for lipid synthesis through reductive carboxylation while maintaining the citrate levels along with overloaded or defective mitochondria (Jiang et al., 2016; Mullen et al., 2012; Plaitakis, Kalef-Ezra, Kotzamani, Zaganas, & Spanaki, 2017; Yang, Venneti, & Nagrath, 2017). These nutritional effects together with GDH/ α-KG/fumarate-maintained redox homeostasis (Jin et al., 2015; Niemiec et al., 2011) and α-KG-mediated dietary restriction might help lifespan extension (Asadi Shahmirzadi et al., 2020; Chin et al., 2014; Tian et al., 2020). On the other hand, increased GDH activity could be detected in various brain pathologies including Parkinson’s disease (Bao et al., 2009; Plaitakis et al., 2010), and suppression of GDH extended the lifespan of worms and flies (Reis-Rodrigues et al., 2012; Talbert et al., 2015), further stressing the twofold contributions of GDH/α-KG to aging. Elevated glutaminolysis is also a hallmark of cancer metabolism and balancing ROS generation could promote tumorigenesis (Jin et al., 2015; Yang et al., 2017), therefore, a strategy of SASP inhibition in parallel with the preservation of supportive functionalities of GDH/α-KG in metabolism might optimize the efficacy of GDH modulation in aging intervention and reduce the hazard of metabolic disorders (Ortsater, Grankvist, Wolfram, Kuehn, & Sjoholm, 2012; Pournourmohammadi et al., 2017) and cancer development.

**SUPPLEMENTAL EXPERIMENTAL PROCEDURES**

**Plasmids, siRNAs, and lentiviral transfections**

The human SIRT4 gene was cloned in a pcDNA3.1 vector for transient transfection and HA-tagged pHBLV lentiviral vector for stable expression. Human FOXQ1 was subcloned into the pcDNA3.1-FLAG and pHBLV vector respectively. Deletion mutants of SIRT4 promoter-reporter plasmids were constructed by using oligo-directed, PCR-based mutagenesis. The QuikChange site-directed mutagenesis kit (Stratagene, La Jolla, CA) was used for site-directed mutagenesis. SIRT4 H162Y mutant plasmid was a gift from Prof. Qi-Qun Tang. All clones were confirmed by DNA sequencing. Plasmid transfection was performed using Lipofectamine 2000 Reagent (Invitrogen) and siRNAs were transfected using Lipofectamine RNAi MAX (Invitrogen) Reagent following the manufacturer's protocol. Control siRNA and siRNAs for target genes were synthesized by Genepharm (Shanghai, China). The siRNA sequences were as follows: siSIRT4#1, AGCGGUACUGGGCGAGAAA; siSIRT4#2, GCUUCAUCACCCUUUCCAA; siFOXQ1#1, GACAACUACUGGAUGCUCA; siFOXQ1#2, GCCAAGCAAUUUCUUUAAA; siGDH#1, CAUUGAGAAAGUCUUCAAA; siGDH#2, AAGUGAGUUCUUAGUAUUU; siControl, UUCUCCGAACGUGUCACGU. For lentiviral packaging, HEK293T cells were co-transfected with pMD2G and psPAX2 and the shRNA-pHBLV constructs, and viral supernatant was harvested by filtration after 48h. For infections, 2BS cells were incubated with viral supernatant in the presence of Polybrene (8 μg/ml) to increase the infection efficiency. shRNAs were inserted into the pHBLV vector at EcoRI and BamHI sites using ClonExpress MultiS One Step Cloning Kit (Vazyme, C113-02). Targeting sequences of shSIRT4, shFOXQ1, shGDH and shScramble were: 5’- AGCGGTACTGGGCGAGAAA -3’; 5’- GACAACTACTGGATGCTCA -3’; 5’- AAGTGAGTTCTTAGTATTT -3’; 5’- TTCTCCGAACGTGTCACGT -3’.

**Tissue and homogenate preparation**

The brain was dissected into the three distinct regions: cerebral cortex, hippocampus, and striatum as described by Spijker (Andersen, Jakobsen, Waagepetersen, & Aldana, 2019) and were homogenized (1:10, w/v) in SETH buffer, pH 7.4 (250 mM sucrose, 2 mM EDTA, 10 mM Tris, 50 IU/mL heparin). The homogenates were centrifuged at 800 x g for 10 min at 4 °C and supernatants kept at -80 °C for enzyme activity determination (use within 5 days) or protein analysis by western blotting. Protein content was determined using the BCA Protein Assay Kit (Pierce).

**Immunoprecipitation and western blotting**

For Immunoprecipitation, Dynabeads Protein G (Invitrogen) were pre-incubated with anti-GDH antibody (Proteintech,14299-1-AP) at 4°C overnight with rotation. Cells or tissues were lysed at 4°C in ice-cold NP40 buffer (50 mM Tris [pH 8.0], 150 mM NaCl, 1% NP40) containing 1 mM NaF, 1 mM Na3VO4, 1 mM dithiothreitol (DTT), 1 mM phenylmethylsulfonyl fluoride (PMSF) and protease inhibitor cocktail (Amresco). The lysate was clarified by centrifugation at 13,000 rpm at 4°C for 15 min, immunoprecipitated at 4°C for 2h, washed, and eluted by boiling for 10 min in 2x SDS loading buffer for western blot analysis. TrueBlot (Rockland) as secondary antibodies were used to avoid heavy-/light-chain antibody interference. Western blot was performed as previously described (Masui et al., 2013). Primary antibodies used were as follows: anti-FOXQ1 (Abcam, ab51340 and Santa Cruz, sc-166266 X); anti-SIRT4 (Abcam, ab168679); anti-GDH (Proteintech, 14299-1-AP); anti-GAPDH (Santa Cruz, sc-47724); anti-Tubulin (Santa Cruz, sc-8035); anti-ADP-ribose (Cell Signaling Technology Inc, 83732S); anti-ace-Lys (Cell Signaling Technology Inc, 9441s); anti-p-Ser (Santa Cruz, sc-81514); anti‐p‐Thr (Santa Cruz, sc‐5267); anti-p16 (Santa Cruz, sc‐468), anti-p21 (Proteintech, 10355-1-AP), anti-IL-8 (Proteintech , 27095-1-AP).

**Quantitative reverse transcription PCR (RT-qPCR)**

Total RNA was isolated using a RNeasy Mini kit (Qiagen) according to the manufacturer's instructions. Then 2 μg of RNA were reverse transcribed (RevertAid, Thermo Scientific) and analyzed by RT-qPCR in duplicates using SYBR® Select Master Mix (Invitrogen) on an ABI PRISM 7500 sequence detection system (Applied Biosystems). Primer sequences used are listed as following: *GAPDH*, 5′-CCACCCATGGCAAATTCCATG-3′ (forward) and 5′- TGATGGGATTTCCATTGATGAC-3′ (reverse); *SIRT4*, 5′-GTGGAGGCCATCTGAAACCA-3′ (forward) and 5′-TGCAAGGATGATCCCACCAC-3′ (reverse); *FOXQ1*, 5′-ACGCTGGCGGAGATCAACGAG-3′ (forward) and 5′-AGGTTGTGGCGCACGGAGTT-3′ (reverse); *GDH*, 5′-GTTGGTGAGTCTGATGGGA-3′ (forward) and 5′- CCCATGTTGCAATTTGAAGTC -3′ (reverse); *IL-6*, 5′-TACCCCCAGGAGAAGATTCC-3′ (forward) and 5′-TTTTCTGCCAGTGCCTCTTT-3′ (reverse); *IL-8*, 5′-TAGCAAAATTGAGGCCAAGG-3′ (forward) and 5′-AAACCAAGGCACAGTGGAAC-3′ (reverse); *IL-10*, 5′-AAGACCCAGACATCAAGGC-3′ (forward) and 5′-AAGAAATCGATGACAGCGC-3′ (reverse). Data were analyzed with 2^−ΔΔCt^ value calculation using control genes for normalization.

**Luciferase activity assay**

Luciferase reporters were constructed based on the pGL3 basic vector. Reporter transfection was performed by use of lipofectamine 2000 (Invitrogen). Luciferase activity was measured with a dual luciferase kit (Promega) according to the manufacturer’s protocol.

**Chromatin Immunoprecipitation (ChIP)**

ChIP experiments were performed according to the previously described protocol (Masui et al., 2013). The precipitated DNA was quantified by real-time PCR. The antibodies used in ChIP were as following: anti-FOXQ1 (Santa Cruz, sc-166266 X); anti-H3K9me3 (Abcam, ab8898). Normal rabbit or mouse IgG (Santa Cruz) was used as negative control. The primers used for ChIP-qPCR analysis of *SIRT4* promoter were: 5’-CGGAGGATTGTGGGACTTGT-3’ (forward) and 5’-GGCCTTTTGCTGACCTCTCA-3’ (reverse). The primers used for ChIP-qPCR analysis of *IL-6*/*IL-8* promoters were reported previously (Raskatov et al., 2012).

**Measurement of senescence‐associated phenotypes**

CCK-8, EdU incorporation and SA-β-gal staining experiments were performed according to the previously described protocols (Fu et al., 2018; Masui et al., 2013).

**Nuclei isolation for metabolite measurements**

Isolation of nuclei was performed using the commercially available nuclei isolation kit: Nucl-Cyto-Mem preparation kit (Applygen Technologies Inc., Beijing, China) according to the manufacturer’s instruction. In brief, the 2BS cells were homogenated in CER, the lysates were then centrifuged at 1000 g for 5 min. The precipitates were suspended and washed with NER, and centrifuged at 4000 g for 5 min, discarded the supernatant, and the nucleic component could be obtained. Nuclei were then prepared for either metabolite measurements (immediately) or protein extraction.

**Analyses of metabolites and enzymatic activities**

The intracellular levels of α-KG and succinate were determined by using α-ketoglutarate assay kit (Biovision，#K677-100) and succinate colorimetric assay kit (Sigma-Aldrich, MAK184) following the manufacturer’s instructions. The enzymatic activity of GDH and CS were measured using GDH activity assay kit (Sigma-Aldrich, MAK099) and Citrate Synthase Activity Assay Kit (Cayman Chemical, 701040) according to the manufacturer's instructions. Metabolites and enzymatic activities were normalized to protein concentration. Enzyme activity was expressed as units mU/μg (nmol substrate converted per minute per microgram protein).

**SUPPLEMENTAL REFERENCES**

Andersen, J. V., Jakobsen, E., Waagepetersen, H. S., & Aldana, B. I. (2019). Distinct differences in rates of oxygen consumption and ATP synthesis of regionally isolated non-synaptic mouse brain mitochondria. *J Neurosci Res, 97*(8), 961-974. doi:10.1002/jnr.24371

Asadi Shahmirzadi, A., Edgar, D., Liao, C.-Y., Hsu, Y.-M., Lucanic, M., Asadi Shahmirzadi, A., . . . Lithgow, G. J. (2020). Alpha-Ketoglutarate, an Endogenous Metabolite, Extends Lifespan and Compresses Morbidity in Aging Mice. *Cell Metab, 32*(3), 447-456.e446. doi:<https://doi.org/10.1016/j.cmet.2020.08.004>

Bao, X. D., Pal, R., Hascup, K. N., Wang, Y. F., Wang, W. T., Xu, W. H., . . . Michaelis, E. K. (2009). Transgenic Expression of Glud1 (Glutamate Dehydrogenase 1) in Neurons: In Vivo Model of Enhanced Glutamate Release, Altered Synaptic Plasticity, and Selective Neuronal Vulnerability. *Journal of Neuroscience, 29*(44), 13929-13944. doi:10.1523/Jneurosci.4413-09.2009

Chin, R. M., Fu, X., Pai, M. Y., Vergnes, L., Hwang, H., Deng, G., . . . Huang, J. (2014). The metabolite α-ketoglutarate extends lifespan by inhibiting ATP synthase and TOR. *Nature, 510*(7505), 397-401. doi:10.1038/nature13264

Cruzat, V., Macedo Rogero, M., Noel Keane, K., Curi, R., & Newsholme, P. (2018). Glutamine: Metabolism and Immune Function, Supplementation and Clinical Translation. *Nutrients, 10*(11). doi:10.3390/nu10111564

Fu, J., Huang, D., Yuan, F., Xie, N., Li, Q., Sun, X., . . . Zhang, Y. (2018). TRAF-interacting protein with forkhead-associated domain (TIFA) transduces DNA damage-induced activation of NF-kappaB. *Journal of Biological Chemistry, 293*(19), 7268-7280. doi:10.1074/jbc.RA117.001684

Jiang, L., Shestov, A. A., Swain, P., Yang, C., Parker, S. J., Wang, Q. A., . . . DeBerardinis, R. J. (2016). Reductive carboxylation supports redox homeostasis during anchorage-independent growth. *Nature, 532*(7598), 255-258. doi:10.1038/nature17393

Jin, L., Li, D., Alesi, G. N., Fan, J., Kang, H. B., Lu, Z., . . . Kang, S. (2015). Glutamate dehydrogenase 1 signals through antioxidant glutathione peroxidase 1 to regulate redox homeostasis and tumor growth. *Cancer Cell, 27*(2), 257-270. doi:10.1016/j.ccell.2014.12.006

Masui, K., Tanaka, K., Akhavan, D., Babic, I., Gini, B., Matsutani, T., . . . Mischel, P. S. (2013). mTOR complex 2 controls glycolytic metabolism in glioblastoma through FoxO acetylation and upregulation of c-Myc. *Cell Metab, 18*(5), 726-739. doi:10.1016/j.cmet.2013.09.013

Mullen, A. R., Wheaton, W. W., Jin, E. S., Chen, P.-H., Sullivan, L. B., Cheng, T., . . . DeBerardinis, R. J. (2012). Reductive carboxylation supports growth in tumour cells with defective mitochondria. *Nature, 481*(7381), 385-388. doi:10.1038/nature10642

Niemiec, T., Sikorska, J., Harrison, A., Szmidt, M., Sawosz, E., Wirth-Dzieciolowska, E., . . . Pierzynowski, S. (2011). Alpha-ketoglutarate stabilizes redox homeostasis and improves arterial elasticity in aged mice. *J Physiol Pharmacol, 62*(1), 37-43.

Ortsater, H., Grankvist, N., Wolfram, S., Kuehn, N., & Sjoholm, A. (2012). Diet supplementation with green tea extract epigallocatechin gallate prevents progression to glucose intolerance in db/db mice. *Nutr Metab (Lond), 9*, 11. doi:10.1186/1743-7075-9-11

Plaitakis, A., Kalef-Ezra, E., Kotzamani, D., Zaganas, I., & Spanaki, C. (2017). The Glutamate Dehydrogenase Pathway and Its Roles in Cell and Tissue Biology in Health and Disease. *Biology (Basel), 6*(1). doi:10.3390/biology6010011

Plaitakis, A., Latsoudis, H., Kanavouras, K., Ritz, B., Bronstein, J. M., Skoula, I., . . . Spanaki, C. (2010). Gain-of-function variant in GLUD2 glutamate dehydrogenase modifies Parkinson's disease onset. *European Journal of Human Genetics, 18*(3), 336-341. doi:10.1038/ejhg.2009.179

Pournourmohammadi, S., Grimaldi, M., Stridh, M. H., Lavallard, V., Waagepetersen, H. S., Wollheim, C. B., & Maechler, P. (2017). Epigallocatechin-3-gallate (EGCG) activates AMPK through the inhibition of glutamate dehydrogenase in muscle and pancreatic ss-cells: A potential beneficial effect in the pre-diabetic state? *Int J Biochem Cell Biol, 88*, 220-225. doi:10.1016/j.biocel.2017.01.012

Raskatov, J. A., Meier, J. L., Puckett, J. W., Yang, F., Ramakrishnan, P., & Dervan, P. B. (2012). Modulation of NF-kappa B-dependent gene transcription using programmable DNA minor groove binders. *Proceedings of the National Academy of Sciences of the United States of America, 109*(4), 1023-1028. doi:10.1073/pnas.1118506109

Reis-Rodrigues, P., Czerwieniec, G., Peters, T. W., Evani, U. S., Alavez, S., Gaman, E. A., . . . Hughes, R. E. (2012). Proteomic analysis of age-dependent changes in protein solubility identifies genes that modulate lifespan. *Aging Cell, 11*(1), 120-127. doi:10.1111/j.1474-9726.2011.00765.x

Talbert, M. E., Barnett, B., Hoff, R., Amella, M., Kuczynski, K., Lavington, E., . . . Eanes, W. F. (2015). Genetic perturbation of key central metabolic genes extends lifespan in Drosophila and affects response to dietary restriction. *Proceedings of the Royal Society B-Biological Sciences, 282*(1815). doi:ARTN 20151646

10.1098/rspb.2015.1646

Tian, Q., Zhao, J., Yang, Q., Wang, B., Deavila, J. M., Zhu, M.-J., & Du, M. (2020). Dietary alpha-ketoglutarate promotes beige adipogenesis and prevents obesity in middle-aged mice. *Aging Cell, 19*(1), e13059. doi:10.1111/acel.13059

Wu, N., Yang, M., Gaur, U., Xu, H., Yao, Y., & Li, D. (2016). Alpha-Ketoglutarate: Physiological Functions and Applications. *Biomol Ther (Seoul), 24*(1), 1-8. doi:10.4062/biomolther.2015.078

Yang, L., Venneti, S., & Nagrath, D. (2017). Glutaminolysis: A Hallmark of Cancer Metabolism. *Annu Rev Biomed Eng, 19*, 163-194. doi:10.1146/annurev-bioeng-071516-044546
