# Supplementary material for: Epigenetic activation of secretory phenotypes in senescence by the FOXQ1-SIRT4-GDH signaling
Source: Cell Death Dis. 2023 Jul 29;14(7):481. doi: 10.1038/s41419-023-06002-9 (PMC10387070; doi:10.1038/s41419-023-06002-9)

Original gel for western blotting

Figure 1D

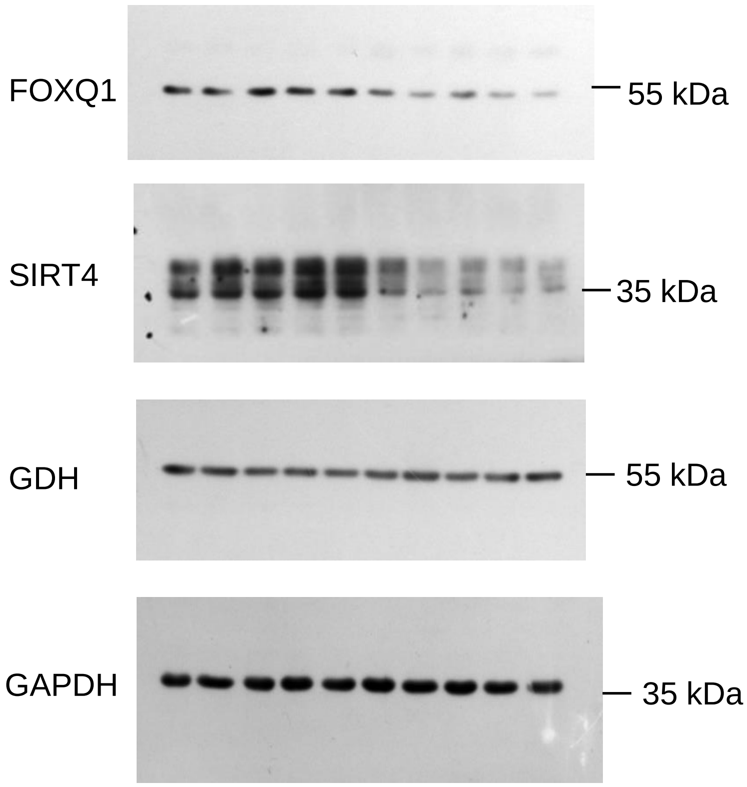

Figure 1E

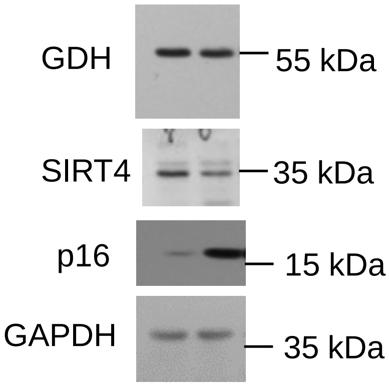

Figure 1F

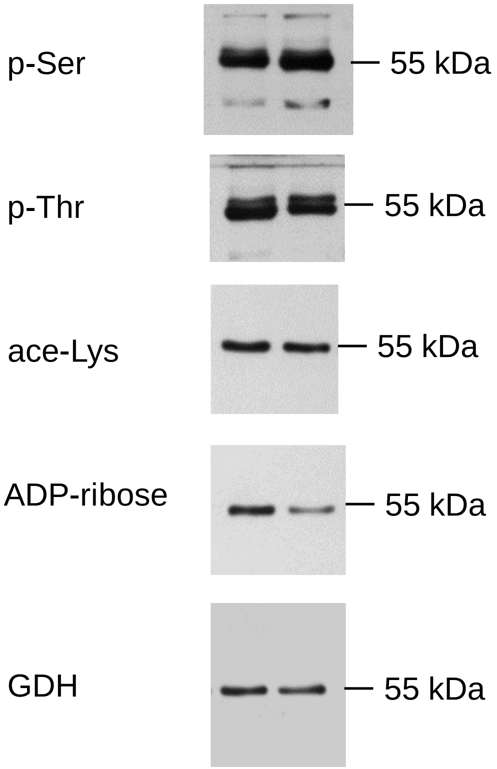

Figure 1G and 1H

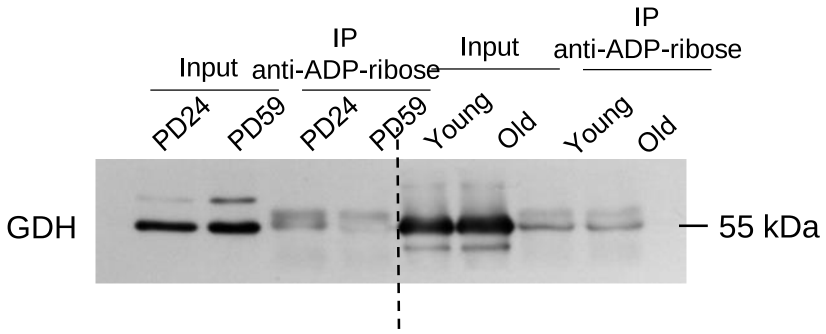

Figure 2A

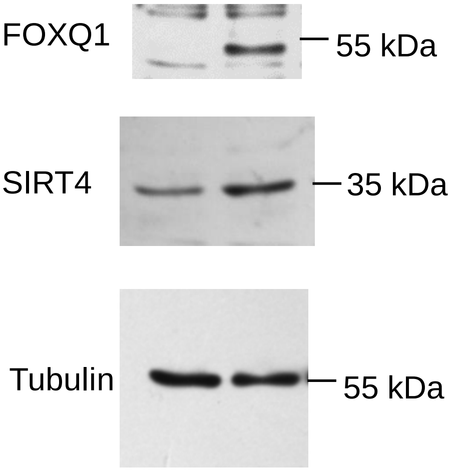

Figure 2B

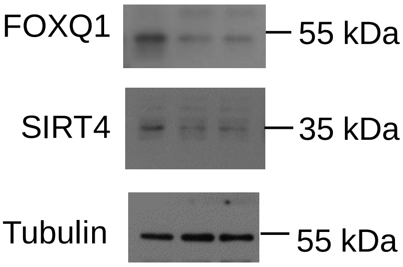

Figure 3A

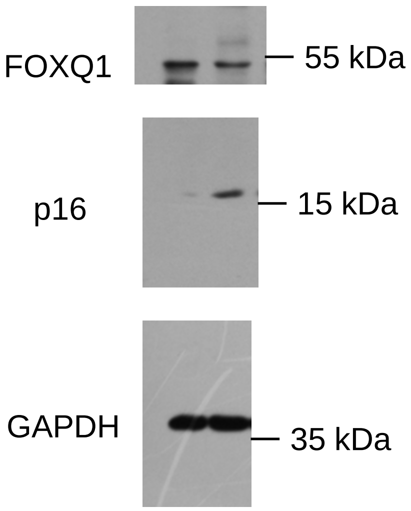

Figure 3C

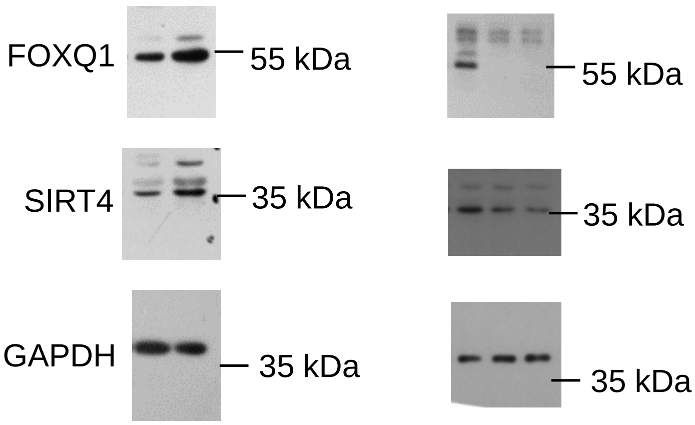

Figure 4D

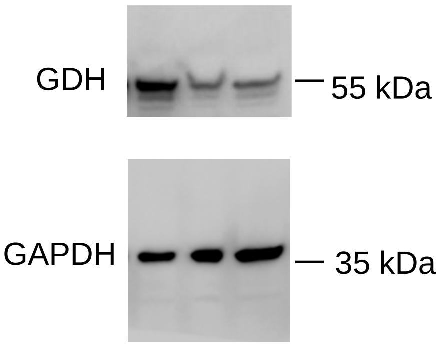

Supplemental Figure 1F

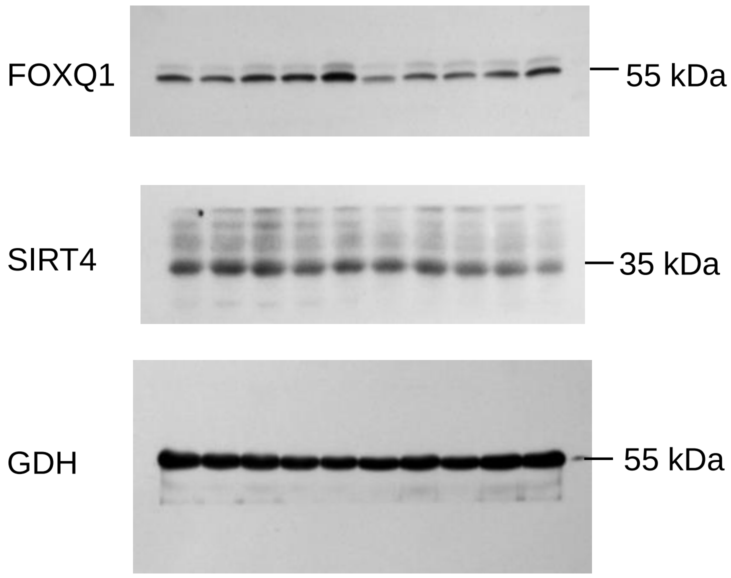

Supplemental Figure 1G

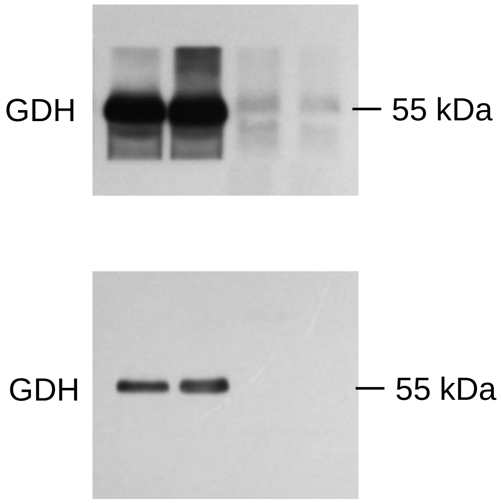

Supplemental Figure 1H

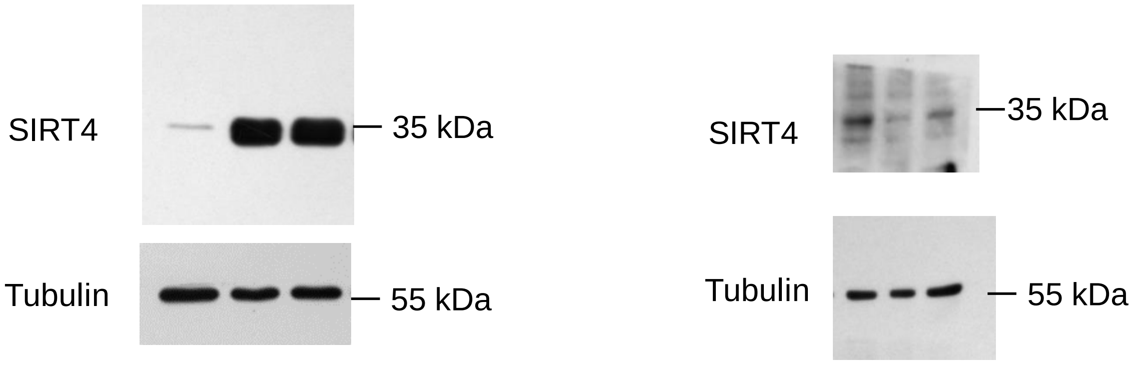

Supplemental Figure 2A

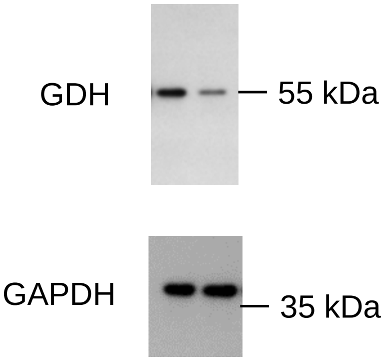

Supplemental Figure 2N

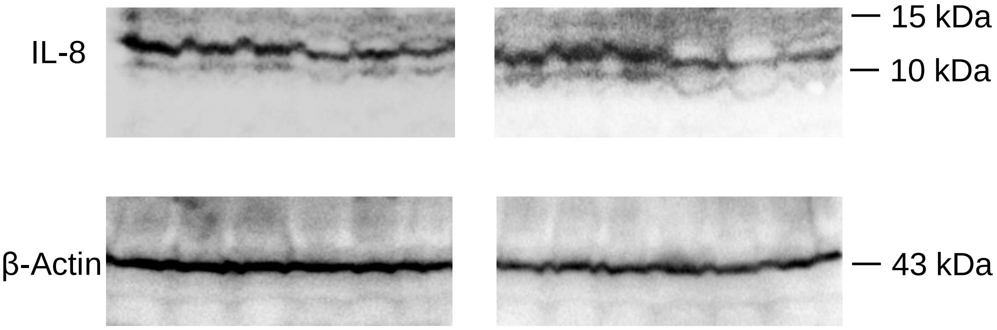

Supplemental Figure 2R

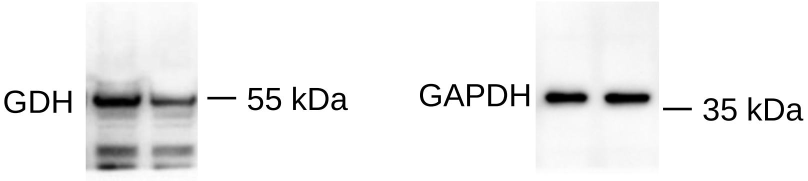

Supplemental Figure 4A

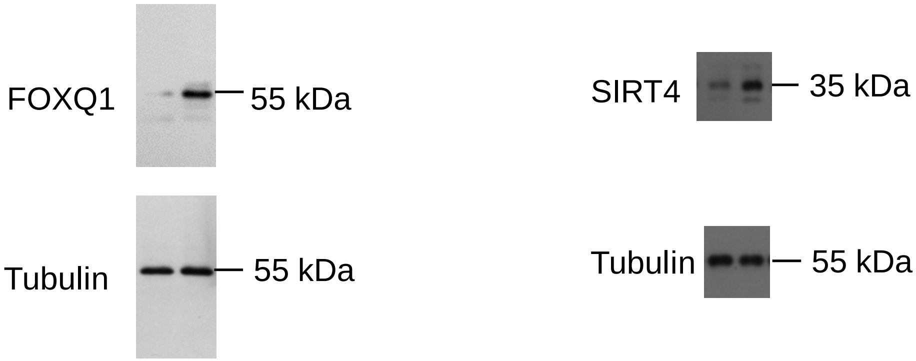

Supplemental Figure 4B

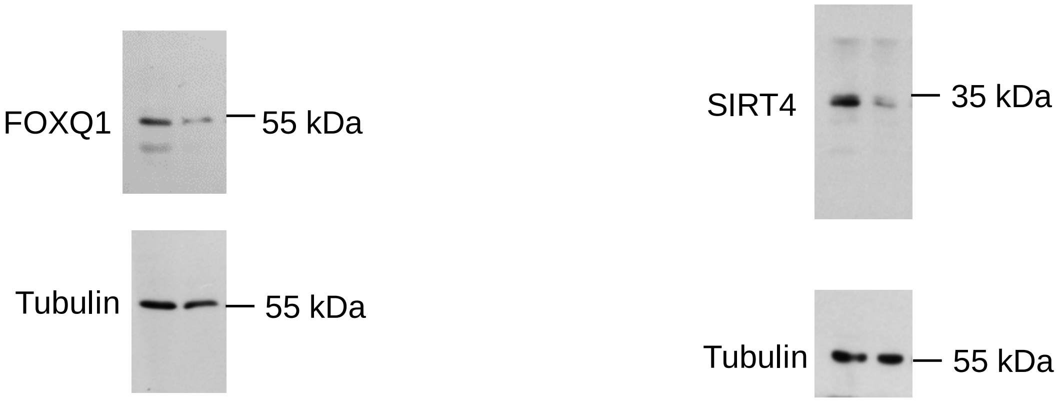

Supplemental Figure 4C

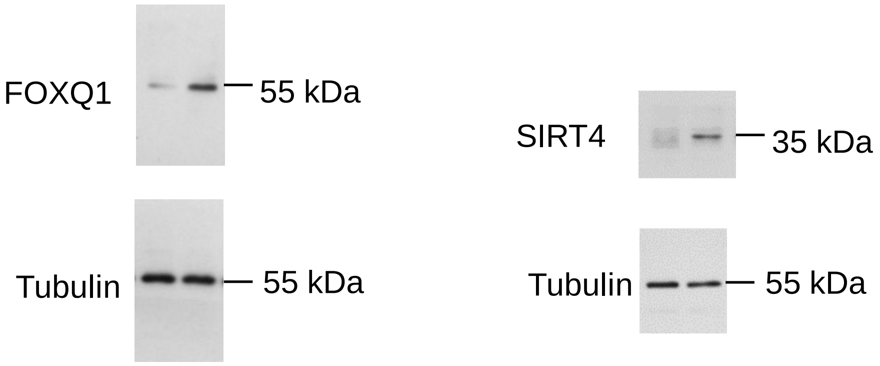

Supplemental Figure 4D

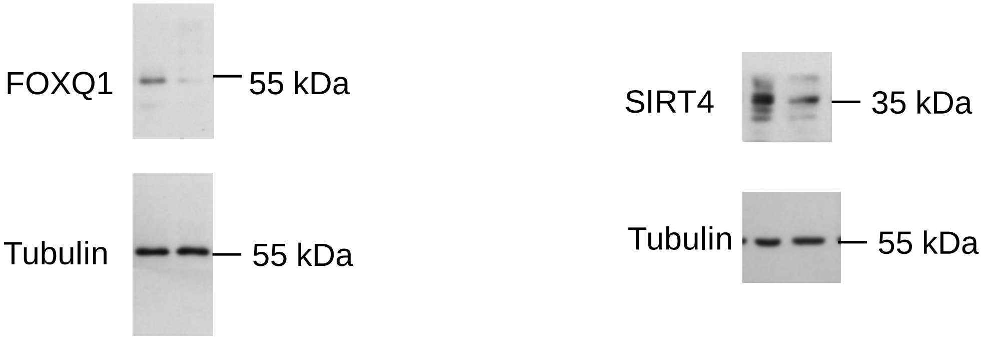

Supplemental Figure 4E

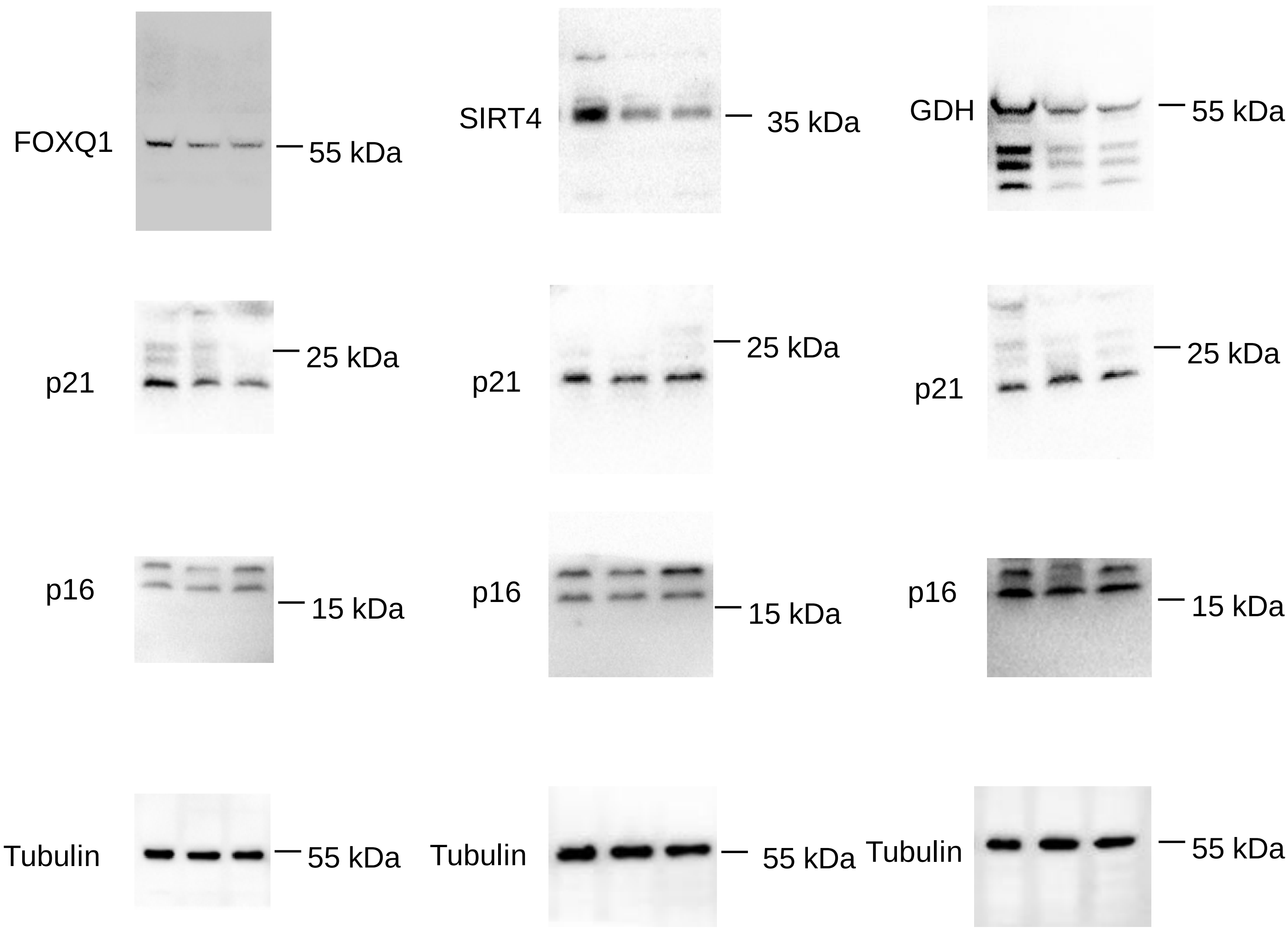

Supplement: Supplementary file 4 — Original Data File [file 41419_2023_6002_MOESM4_ESM.pdf]
